# Associations between biomarkers and skeletal muscle function in individuals with osteoarthritis: a systematic review and meta-analysis

**DOI:** 10.1186/s13075-024-03419-1

**Published:** 2024-11-05

**Authors:** Stephanie L. Smith, Lorna Paul, Martijn P. M. Steultjens, Rebecca L. Jones

**Affiliations:** 1 Pain Centre Versus Arthritis, Advanced Pain Discovery Platform, University of Nottingham, Nottingham, UK; 2 Academic Rheumatology, Division of Injury, Recovery and Inflammation Sciences, School of Medicine, University of Nottingham, Nottingham, UK; 3 NIHR Nottingham Biomedical Research Centre, University of Nottingham, Nottingham, UK; 4 Research Centre for Health (ReaCH), School of Health and Life Sciences, Glasgow Caledonian University, Glasgow, UK; 5 Health Advancement Research Team (HART), School of Sport and Exercise Science, University of Lincoln, Lincoln, UK

**Keywords:** Lower limb, Biochemical markers, Muscle strength, Inflammation, Genetics, Metabolic, Biological markers, Function, Disability

## Abstract

**Objectives:**

Skeletal muscle dysfunction is the primary cause of functional limitations in osteoarthritis, associated biomarkers have the potential as targets for early disease identification, diagnosis, and prevention of osteoarthritis disability. This review aimed to identify associations between biomarkers and lower limb skeletal muscle function in individuals with osteoarthritis.

**Methods:**

A systematic literature review and meta-analysis conducted in PubMed, MEDLINE, CINAHL, EMBASE, Scopus, SPORTDiscus and Web of Science databases from inception to 8^th^ August 2023. Two independent reviewers performed the title, abstract, full-text screening, data extraction and methodological quality assessment. A meta-analysis was undertaken based on the available data.

**Results:**

Twenty-four studies with 4101 participants with osteoarthritis were included (females: 78%; age range; 49 to 71 years). One study reported muscle-specific biomarkers (*n* = 3), whilst six studies reported osteoarthritis-specific markers (*n* = 5). Overall, 93 biomarkers were reported, predominately characterised as inflammatory (*n* = 35), metabolic (*n* = 15), and hormones (*n* = 10). Muscle strength and vitamin D reported a significant association (Hedge’s *g*: 0.58 (Standard Error (SE): 0.27; *P* = 0.03), *k* = 3 studies). Walking speed and high-sensitivity C-reactive protein reported no significant associations (Hedge’s *g*: -0.02 (SE: 0.05; *P* = 0.73), *k* = 3 studies).

**Conclusion:**

Associations between biomarkers and lower limb skeletal muscle function in individuals with osteoarthritis was limited, the few studies exploring lower limb muscle measures were mainly secondary outcomes. Furthermore, biomarkers were largely related to overall health, with a lack of muscle specific biomarkers. As such, the mechanistic pathways through which these associations occur are less evident, and difficult to draw clear conclusions on these relationships.

**Trial registration:**

Registered on PROSPERO (CRD42022359405).

**Supplementary Information:**

The online version contains supplementary material available at 10.1186/s13075-024-03419-1.

## Background

Osteoarthritis (OA) is a heterogeneous condition with a complex multifactorial pathogenesis driving different outcomes and is one of the leading causes of pain and disability worldwide [1]. Finding effective disease- and symptom-modifying therapies is a global unmet need. Yet, effective therapies remain elusive, predominantly due to the inability to detect early OA but also due to poor measures of progression [2]. Diagnosis of OA is currently based on radiographic criteria and clinical symptoms [3] with evidence evaluating new OA treatments also based on these measures. Imaging modalities and patient-reported outcome measures fail to detect molecular changes, which can proceed the morphological changes they detect [4]. Biomarkers from blood, urine, and synovial fluid objectively measure and evaluate indicators of normal biological processes, pathogenic processes, or pharmacological responses to therapeutic interventions. Therefore, these markers have the potential to reflect and quantify changes and overcome some of the limitations of current methods for OA assessment [5].

Currently, there is particular interest in the use of biomarkers for the diagnosis, monitoring, evaluation, and prediction of OA treatment response [6, 7], with a growing body of systematic reviews of markers of OA [8, 9]. The primary aim of these biomarkers is OA diagnosis and prevention. As such biomarkers including circulating inflammatory markers [10] and hormones [11, 12] (e.g., leptin, insulin-like growth factor-1 (IGF-1)), have been identified and associated with changes in skeletal muscle function.

Skeletal muscle function has been implicated as a risk factor for the incidence and progression of OA [13], and disability [13], such as mobility difficulties (e.g., walking, climbing stairs) and falls. Mobility difficulties are known to have detrimental effects on an individual’s ability to live independently and their quality of life [14], also leading to falls, disability and subsequent adverse health outcomes [15]. As such, identifying biomarkers associated with skeletal muscle function could aid in the early diagnosis, treatment and prevention of OA and OA-related disability through the development of targeted treatments.

Despite the high prevalence of OA, and the emergence of potential biomarkers as a tool to aid diagnosis and treatment, lower limb skeletal muscle dysfunction is often overlooked, despite its critical role in the disease process and outcomes. Whilst muscle strength is easily detected in clinical practice, biomarkers of muscle which detect the molecular changes preceding functional decline is essential not only as potential targets for early disease identification and diagnosis but for prevention of OA-related disability. Currently, research is progressing in terms of the identification of prognostic biomarkers, with an extensive variety of biomarkers and measures of lower limb muscle function. Synthesis is required to understand inconsistent results, understand all, if any, associations, and identify biomarkers as indicators of skeletal muscle dysfunction in people with osteoarthritis following targeted interventions. Accordingly, the present systematic review and meta-analysis aimed to identify associations between biomarkers and lower limb skeletal muscle function in individuals with OA.

## Methods

The current review protocol was designed in accordance with the Preferred Reporting Items for Systematic Reviews and Meta-Analyses (PRISMA) [16] and registered on PROSPERO (CRD42022359405).

### Search strategy

A systematic search to identify associations between biomarkers and lower limb skeletal dysfunction was conducted in eight databases (PubMed, AMED, CINAHL, EMBASE, MEDLINE, Scopus, SPORTDiscus, Web of Science). A unique systematic block search of Boolean terms was developed in PubMed was implemented in four blocks (biological marker, osteoarthritis, lower limb and performance outcome) and replicated as closely as possible in the other databases (Supplementary Table 1) from inception to 8^th^ August 2023. The reference list from identified studies and relevant reviews was also undertaken to identify any further studies and were added to full-text screening manually.

### Selection criteria

English language original articles published in peer-reviewed journals were included. Review articles, conferences abstracts, and grey literature were excluded. Searchers were imported into Covidence (Covidence systematic review software, Veritas Health Innovation, Melbourne, Australia) for eligibility screening. Population, Intervention, Comparator, Outcomes and Study design (PICOS; Table 1) was used to define inclusion and exclusion criteria. Individuals were required to be all, or a distinct subgroup of adults (aged > 18 years) diagnosis/classification of OA. All definitions of osteoarthritis were included. Knee and hip OA were both included due to their similarities in muscle dysfunction patterns (e.g., atrophy, muscle inhibition, reduced quality) [17] and higher prevalence of sarcopenia compared to individuals without hip or knee OA [18]. Only original peer-reviewed studies examining the relationship between biological markers (biomarkers), and measurement of lower limb skeletal muscle function (e.g., muscle strength, mass, function) were included. Following duplicate removal, a two-phase screening strategy 1) title and abstract, 2) full-text appraisal) was conducted by two independent reviewers (SLS and RLJ). Discrepancies were resolved by discussion, where consensus was not achieved a third reviewer (LP) was consulted.

**Table 1:** Population, intervention or exposure, comparator, outcomes and study design (PICOS) criteria

Population	Individuals were required to be human adults (aged > 18 years) with all, or a distinct subgroup of participants diagnosis/classification of osteoarthritis
Intervention or Exposure	Individuals or a distinct subgroup of individuals were required to have a diagnosis/classification of osteoarthritis. All definitions of osteoarthritis were included within this review. Studies including at-risk population without a diagnosis/classification of osteoarthritis were excluded
Comparator	Examining the relationship between biological markers (biomarkers), and measurement of lower limb muscle function (e.g., muscle strength, mass, power)
Outcomes	Report on a biomarker (defined as a characteristic that is objectively measured and evaluated as an indicator of normal biological processes, pathogenic processes, or pharmacologic responses to a therapeutic intervention [19], excluding imaging-based biomarkers) and measurement of lower limb muscle function regardless of measurement modality
Study design	Only original peer-reviewed research articles in English language were included, with review articles, conferences abstracts, and grey literature excluded. Any study design that included the information described above was considered for inclusion

### Risk of bias

Two reviewers (SLS, RLJ) assessed methodological quality using the Joanna Briggs Institute checklist for analytical cross-sectional studies [20] due to the extraction of only baseline data, treating all studies as cross-sectional. Each criterion was recorded as ‘Yes’, ‘No’, ‘Unclear’, ‘Not applicable’, and overall determined ‘Include’, ‘Exclude’, ‘Seek further information’ (Table 2). If more than 50% of items were recorded as ‘No’ or ‘Unclear’ papers were considered high risk of bias [21]. Papers susceptible to high risk of bias were excluded to reduce bias in the study findings [22].

**Table 2:** Check list for study quality from Joanna Briggs Institute for Cross-sectional studies; scoring Yes =  + , No = -, unsure = ?

Author	Were the criteria for inclusion in the study clearly defined?	Were the study subjects and the setting described in detail?	Was the exposure measured in a valid and reliable way?	Were objective; standard criteria used for measurement of the condition	Were confounding factors identified?	Were strategies to deal with confounding factors stated	Were the outcomes measured in a valid and reliable way?	Was appropriate statistical analysis used?	Overall appraisal
Barker, et al. [23]	+	+	+	+	-	-	+	+	**Include**
Chang, et al. [24]	+	-	+	+	-	-	+	+	**Include**
Durmus, et al. [11]	+	?	+	+	-	-	+	-	**Include**
El-Fetiany, et al. [25]	+	-	+	+	-	-	+	+	**Include**
Glover, et al. [26]	+	+	-	-	-	-	+	-	**Exclude**
Gökçen, et al. [27]	+	?	+	+	-	-	+	+	**Include**
Herrero-Manley, et al. [28]	+	+	+	+	-	-	+	+	**Include**
Hunt, et al. [29]	+	-	+	+	+	+	+	+	**Include**
Javadian, et al. [30]	+	+	+	+	+	+	+	+	**Include**
Koeckhoven et al. [31]	+	+	+	+	+	+	+	+	**Include**
Kurita, et al. [18]	+	+	+	+	+	+	+	+	**Include**
Levinger, et al. [32]	+	-	+	-	-	-	+	+	**Include**
Levinger, et al. [33]	+	-	+	-	+	+	+	+	**Include**
Levinger, et al. [34]	+	?	+	-	-	-	+	+	**Include**
Manoy, et al. [35]	+	-	+	+	+	+	+	+	**Include**
Manoy, et al. [36]	+	+	+	+	+	+	+	+	**Include**
Miller, et al. [37]	+	-	+	-	+	+	+	+	**Include**
Miller, et al. [38]	+	-	+	-	+	+	+	+	**Include**
Pagura, et al. [12]	+	?	+	-	+	+	+	+	**Include**
Penninx, et al. [39]	+	+	+	+	+	+	+	+	**Include**
Sakr, et al. [40]	+	+	+	+	-	-	+	+	**Include**
Sanchez-Ramirez, et al. [41]	+	-	+	+	+	+	+	+	**Include**
Santos, et al. [42]	+	-	+	+	-	-	+	+	**Include**
Selistre, et al. [43]	+	-	+	+	+	+	+	+	**Include**
Udomsinprasert, et al. [44]	+	-	+	+	+	+	+	+	**Include**

### Data extraction

Two independent authors (SLS, RLJ), verified by a third (LP) extracted data using a standardised piloted data extraction form. Data extracted included: author and year; country of origin; study design; sex; age; OA diagnosis criteria (e.g., Kellgren and Lawrence grade (K&L)), location (e.g., knee), pain severity; biomarkers and lower limb skeletal muscle measures. Biomarkers were categorised based on their primary role. Data were extracted as mean, standard deviation, median, interquartile ranges, standard errors and the most adjusted correlations or regression coefficients of associations between skeletal muscle measure and biomarkers. Corresponding authors were contacted by email where data was missing, not reported or additional information was required. None provided additional information and were excluded from the analysis.

### Evidence synthesis

A minimum of three studies reporting the same biological marker and skeletal muscle measure, were pooled for meta-analysis. Where standard deviation (SD) was not provided, SD was estimated from standard error (SE) or 95% confidence interval (95%CI). Standardised mean difference (SMD) and Hedge’s* g* effect size (SE; Standard error) and their corresponding 95%CI were calculated for each outcome for papers that provided unadjusted mean and SD. Hedge’s* g* effect sizes of 0.2, 0.5 and 0.8 were considered small, moderate, and large, respectively [45]. A random effect meta-analysis was conducted on Jamovi (Version 1.6, Sydney, Australia). Statistical heterogeneity was assessed as low (≥ 30%) moderate (≥ 50%) or high-level (≥ 75%) heterogeneity using the I^2^ statistic [46]. High heterogeneity was also indicated from the pooled data with a Q statistic of *p* ≤ 0.05. Publication bias was evaluated by visually inspecting the funnel plot; this approach was selected due to the lower reliability and statistical power of the Egger’s Regression Test due when dealing with fewer than 10 studies [17]. Data is reported as Hedge’s *g* effect sizes, with positive values indicating a greater association between lower limb muscle measure and the biological marker. Statistical significance was accepted at *P* ≤ 0.05.

## Results

**Fig. 1:**
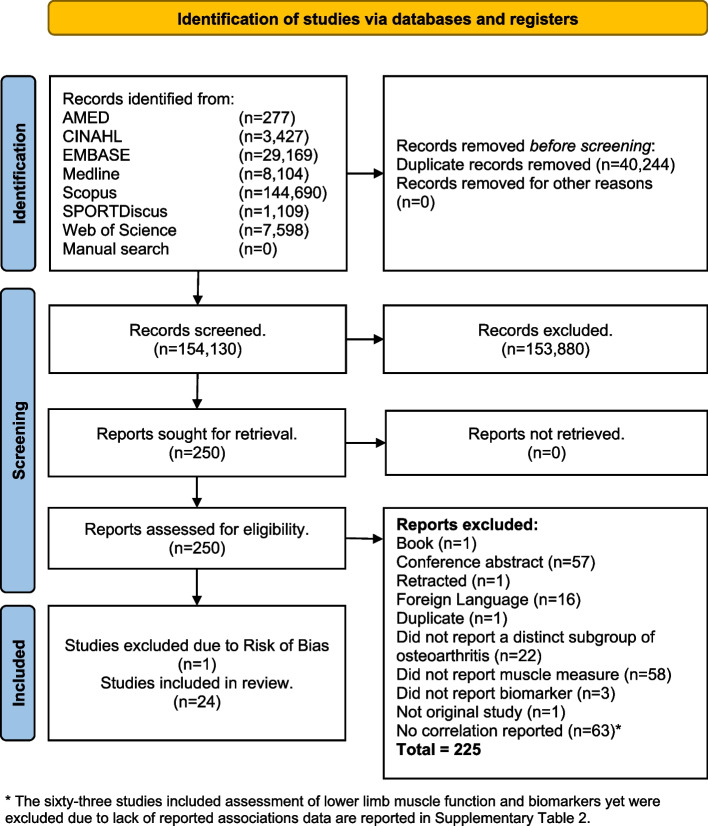
Flow diagram of the study selection process for eligible studies in the systematic review

The study selection process is shown in Fig. 1. Of the 225 studies excluded, 63 studies included assessment of lower limb muscle function and biomarkers yet did not report associations (Supplementary Table 2). Twenty-five articles meet the inclusion criteria. One study [26] was excluded based on risk of bias assessment, five of the eight methodological quality areas highlighting the possibility of bias in its design, conduct and analysis (Table 2). Of the remaining 24 studies, the most frequent risk of bias was the lack of confounders being identified and dealt with. Overall agreement on risk of bias between reviewers was 93%.

### Study characteristics

A total of 4852 participants were included across 24 studies (Table 3), 4101 participants had OA (751 controls), and 78% (*n* = 3,191) of the OA population were female. Two studies were female only [11, 42], 22 were mixed sex, two stratified by sex [12], whilst one included a 100% female sarcopenic obesity group [47]. The lowest and highest mean age reported was 49 ± 2 years [23], and 71 ± 5 years [42] respectively. Twenty-three studies reported OA at the knee, with one study reporting knee or hip OA [18]. OA classification was predominately based on radiographic criteria [12, 18, 23–25, 39–43, 48, 49], American College of Rheumatology (ACR) classification [11, 31, 35, 36, 44, 47] or a combination [27, 29, 30]. K&L scores varied with 14 studies including early OA (0–1) [28] to moderate and severe OA (2–4) [40]. Eight studies were randomised controlled trials, nine observational, nine cross-sectional and one case–control study. Lower limb skeletal muscle measures predominantly included strength [23, 27, 30–32, 34–36, 41, 42, 44, 47, 48], and, function (e.g., gait speed, get-up and go, chair stand, stair negotiation) [12, 18, 24–26, 28, 33, 35–40, 43, 44, 47, 49, 50] tests. Biomarkers identified were classified as inflammatory (*n* = 35), metabolic (*n* = 15), and hormones (*n* = 10), oxidative stress (*n* = 9), bone (*n* = 9), enzyme (*n* = 6), genetic (*n* = 4), muscle (*n* = 3), vitamin (*n* = 1) and glycoprotein (*n* = 1). A limited number of muscle or OA-specific markers were identified in the review. One study found no association between gait speed and muscle-specific biomarkers (creatine phosphokinase, aspartate aminotransferase, alanine aminotransferase) [18]. Whilst six studies identified associations between OA-specific biomarkers (tumour necrosis factor alpha (TNF-a), interleukin 1 (IL-1), c-terminal telopeptide type II collagen (CTX-II), cleavage of type ii collagen by collagenases (C2C), cartilage oligomeric matrix protein (COMP)) [23, 27, 29, 32, 39, 43] with mixed results. Two studies found significant associations between muscle strength and TNF-α [23, 32], and no significant association with CTX-II [27, 43].

**Table 3:** Study characteristics of papers (*n* = 25), including lower limb muscle measure and a biological marker

Author	Study Design	Total sample size	Osteoarthritis subgroup	Osteoarthritis subgroup sample size (M/F)	Age (years)	Diagnosis criteria	Osteoarthritis location	K&L Grade	ACR	Lower limb muscle measure	Biological marker
Barker, et al. [23]	Cross-sectional	29	Whole group	29	49 ± 2	Radiographic	Knee	> 2	No	Peak isometric force, Peak isometric torque, Peak isokinetic concentric extension torque, Peak isokinetic concentric flexion torque	[Serum]: Cu/ZnSOD, GM-CSF, IFN-γ, IL-10, IL-12, IL-13, IL-1β, IL-2, IL-4, IL-5, IL-6, IL-7, IL-8, Mn SOD, IL-1r1, IL-1r2, IL-4r, IL-6r, sTNFr-1, sTNFr-2, TNF-α
*USA*				(13 / 16)							
Chang, et al. [24]	Case control	200	OA	100	70 ± 9	Radiographic	Knee	> 2	No	ASMI, Handgrip, Dumbbells curls, Leg-back strength, chair-stand test, gait speed, SPPB	[Serum]: TAC, Albumin, BUN, Creatinine, GOT, GPT, hs-CRP, total cholesterol [Plasma]: malondialdehyde, coenzyme Q10, coenzyme Q10/TC, protein carbonyl [Whole blood]: RBC TAC
*Taiwan*				(27 / 73)							
Durmus, et al. [11]	RCT	37	Exercise treatment	19 (0 / 19)	57 ± 6	ACR	Knee	1 to 3	Yes	Knee MVIC extension, 6MWT	[Plasma]: Leptin
*Turkey*			Glucosamine Sulfate	18 (0 / 18)	58 ± 7						
El-Fetiany, et al. [25]	Observational	90	Osteoarthritis	60	54 ± 8	Radiographic	Knee	1 to 4	Yes	Chair stand test, Stair climbs test, 6MWT	[Plasma]: Basic fibroblast growth factor
*Egypt*				(12 / 48)							
Glover, et al. [26]*	Cross-sectional	256	Whole group	(95/161)	57 ± 7	ACR clinical criteria	Knee	Not assessed	Yes	SPPB	[Plasma]: 25(OH)D
*USA*											
Gökçen, et al. [27]	Cross-sectional	152	Whole group	152	57 ± 8	Radiographic and ACR	Knee	Assessed	Yes	Quad isometric muscle strength, Knee isometric extensor torque, Knee isometric flexor torque, Knee isokinetic extensor torque, Knee isokinetic flexor torque	[Plasma]: 25(OH)D [Serum]: CTX-I, CTX-II, Leptin, Osteocalcin
*Turkey*				(22 / 130)							
Herrero-Manley, et al. [28]	RCT	96	Osteoarthritis	48	52 ± 5	Luyten's proposal for early osteoarthritis classification	Knee	0–1	Yes	Gait speed, sit to stand	[Serum]: Total cholesterol, LDL, HDL, CRP, Uric acid, Triglycerides
*Spain*				(9 / 39)							
Hunt, et al. [29]	RCT	17	Whole group	17	66 ± 11	Radiographic and ACR	Knee	2 to 4	Yes	Peak KAM, KAM impulse, Walking speed, Isometric knee extension strength, Isometric knee flexion strength, Isometric hip abduction strength	[Urine]: CTX-II, C2C [Serum]: HA, COMP, CPII, [Mixture]: urinary CTX-II: serum CPII
*Canada*				(8 / 9)							
Javadian, et al. [30]	Cross sectional	92	Whole group	92	50 ± 6	Radiographic and ACR	Knee	1 to 3	Yes	Quadriceps strength	[Serum]: 25(OH)D
*Iran*				(20 / 72)							
Koeckhoven et al. [31]	Observational	319	Whole group	319	60 ± 8	ACR	Knee	0 to 4	Yes	Total muscle strength, Extension, Flexion	[Serum]: 25(OH)D, Creatinine
*Netherlands*				(107 / 212)							
Kurita, et al. [18]	Cross-sectional	1425	Whole group	1425	70 ± 9	Radiographic	Knee and hip	Knee: 1–4	Yes	Gait speed, ASMI	[Serum]: ALT, AST, CK, CRP
*Japan*				(286 / 1139)				Hip: 3–4			
Levinger, et al. [32]	Observational	33	Osteoarthritis	19	70 ± 7	Awaiting knee replacement surgery	Knee	Not reported	No	Knee extensor strength	[Muscle]: MCP-1, Atrogen messenger RNA, Atrogin-1, IL-1β, IL-6, IL-8, JNK1/2, NF kB, p65, STAT-3, TNF- α
*Australia*				(9 / 10)							
Levinger, et al. [33]	Observational	19	Osteoarthritis	19	70 ± 7	Awaiting knee replacement surgery	Knee	Not reported	No	Gait velocity, Knee early stance ROM, Knee impulse	[Muscle]: IL-6, JNK-1, MCP-1, NF-kB, p65, STAT-3
*Australia*				(9 / 10)							
Levinger, et al. [34]	Observational	29	Osteoarthritis	19	66 ± 1	Awaiting knee replacement surgery	Knee	Not reported	No	Knee extensor muscle	[Muscle]: Fox O1 total protein, FoxO1 mRNA, FoxO1 phosphorylated (ser256), IL-15 mRNA, IL-15 protein, pFoxO1:FoxO1 [Synovial fluid]: IL-15 [Serum]: IL-15
*Australia*				(9 / 10)							
Manoy, et al. [35]	Observational	208	Normal weight	99 (4 / 95)	65 ± 7	ACR	Knee	< 3	Yes	6MWT, Gait speed, Grip strength, Knee extension force, STS, TUG	[Serum]: 25(OH)D, Calcium, fasted glucose, lipid profile, hs-CRP, Leptin, Phosphorus, PTH
Thailand			Obesity	80 (13 / 67)	65 ± 7						
			Sarcopenic obesity	29 (0 / 29)	65 ± 7						
Manoy, et al. [36]	Cross-sectional	262	Osteoarthritis	202	65 ± 5	ACR	Knee	> 3	Yes	6MWT, Gait speed, Grip strength, Knee extension force, STS, TUG	[Whole blood]: Blood leukocyte relative telomere length
*Thailand*				(21 / 181)							
Miller, et al. [37]	RCT	87	Intervention	31 (11 / 20)	70 ± 6	Self-reported physician-diagnosed knee	Knee	Not reported	No	6MWT, Stair climb	[Plasma]: IL-6, TNF-α, sTNFr-1, sTNFr-2, CRP
*USA*			Control	36 (16 / 20)	70 ± 6						
Miller, et al. [38]	RCT	309	Whole group	309	69 ± 7	Symptomatic	Knee	Not reported	No	6MWT	[Serum]: Corticosterone, DHEA, Growth Hormone, Testosterone, SHBG
*USA*				(223 / 86)							
Pagura, et al. [12]	Cross-sectional	139	Males	25(25 / 0)	64 ± 7	Radiographic	Knee	Not reported	No	Fast self-paced walk, Normal self-paced walk, Stair function, timed get up and go	[Serum]: IGF-1
*USA*			Females	33 (0 / 33)	66 ± 7						
Penninx, et al. [39]	Observational	274	Whole group	274	68 ± 6	Radiographic	Knee	Not reported	No	Walking speed	[Serum]: CRP, IL-6, TNFa, IL-6sR, IL-1sR, TNF-sR1, TNF-sR2
*USA*				(77 / 197)							
Sakr, et al. [40]	Observational	162	Egyptian	41(7 / 34)	53 ± 7	Radiographic	Knee	2 to 4	Yes	6MWT, Chair stand test	[Serum]: 25(OH)D, Alkaine phosphatase, ALT, Creatinine, Ionised calcium, Phosphorous, PTH
*Egypt*			Yemeni	41 (7 / 34)	56 ± 8						
Sanchez-Ramirez, et al. [41]	Cross-sectional	285	Whole group	285	62 ± 7	Radiographic	Knee	Not reported	Yes	Knee muscle strength isokinetic	[Serum]: CRP, Erythrocyte sedimentation rate
*Netherlands*				(106 / 179)							
Santos, et al. [42]	Observational	80	Whole group	80	71 ± 5	Radiographic	Knee	Not reported	Yes	Body mass peak torque/body mass Quads 60 degrees, Body mass peak torque/body mass Hamstrings 60 degrees, Body mass peak torque/body mass Quads 180 degrees, Body mass peak torque/body mass Hamstrings 180 degrees, Hamstring/Quads muscular balance ratio 60 degrees, Hamstring/Quads muscular balance ratio 180 degrees	[Plasma]: IL-6
*Brazil*				(0 / 80)							
Selistre, et al. [43]	Cross-sectional	25	Whole group	25	58 ± 5	Radiographic	Knee	2 to 3	No	Gait speed, Knee adduction angular impulse, peak Knee adduction moment, peak Knee flexion moment, Walk test 40 m	[Urine]: CTX-II
*Brazil*				(13 / 12)							
Udomsinprasert, et al. [44]	Cross-sectional	227	Osteoarthritis	175	65 ± 9	ACR	Knee	Not reported	Yes	6MWT, ASMI, Gait speed, Grip strength, Knee extensor force, sit to stand, SMI, TUGT	[Serum]: 25(OH)D, Adiponectin, Calcium, Fasting blood glucose, HDL, HOMA-IR, hs-CRP, IL-6, Insulin, LDL, Phosphorus, PTH, Triglycerides
*Thailand*				(17 / 158)							

*Excluded due to risk of bias

*1RM* 1 repetition maximum, *25(OH)D* Vitamin D, *6MWT* 6-min walk test, *ACR* American College of Rheumatology, *ALT* Alanine aminotransferase, *ASMI* Appendicular Skeletal Muscle Index, *AST* Aspartate aminotransferase, *BUN* Blood urea nitrogen, *C2C* Cleavage of type ii collagen by collagenases, *CK* Creatine Kinase, *CPII* Type II Procollagen C-Propeptide, *COMP* Cartilage oligomeric matrix protein, *hs-CRP* High-sensitivity c-reactive protein, *CRP* C-reactive protein, *CTX-I* C-terminal telopeptide type I collagen, *CTX-II* C-terminal telopeptide type II collagen, *Cu/ZnSOD* Cu/Zn Superoxide Dismutase, *DHEA* Dehydroepiandrosterone, *Fox O1* Forkhead box protein O1, *GM-CSF* Granulocyte–Macrophage Colony-Stimulating Factor, *GOT* Glutamic Oxaloacetic Transaminase, *GPT* Glutamic Pyruvic Transaminase, *HA* Hyaluronic acid, *HDL* High-density lipoprotein, *HOMA-IR* Homeostatic model assessment of insulin resistance, *IFN-γ* Interferon-gamma, *IGF-1* Insulin-like growth factor-1, *IL-1β* Interleukin 1 beta, *IL-1r1* Interleukin 1 receptor 1, *IL-1r2* Interleukin 1 receptor 2, *IL-10* Interleukin 10, *IL-12* Interleukin 12, *IL-13* Interleukin 13, *IL-15* Interleukin 15, *IL-17* Interleukin 17, *IL-18* interleukin 187, *IL-2* Interleukin 2, *IL-4* Interleukin 4, *IL-4r* Interleukin 4 receptor, *IL-5* Interleukin 5, *IL-6* Interleukin 6, *IL-6r* Interleukin 6 receptor, *IL-7* Interleukin 7, *IL-8* Interleukin 8, *JNK* c-Jun N-terminal kinase, *KAM* Knee adduction moment, *LDL* Low-density lipoprotein, *MCP-1* Monocyte Chemoattractant Protein-1, *Mn SOD* Manganese Superoxide Dismutase, *MVIC* Maximum voluntary isometric contraction, *NF kB p65* Nuclear factor NF-kappa-B p65 subunit, *PTH* Parathyroid hormone, *RBC* Red blood cells, *RCT* Randomised controlled trial, *RNA* Ribonucleic acid, *ROM* Range of motion, *SHBG* Sex Hormone Binding Globulin, *STAT-3* Signal Transducer and Activator of Transcription 3, *STS* Sit-to-stand, *sTNFR1* Soluble forms tumour necrosis factor alpha receptor 1, *sTNFR2* Soluble forms tumour necrosis factor alpha receptor 2, *TAC* Total antioxidant capacity, *TNF-α* Tumour necrosis factor alpha, *TUG* Timed-up and go

### Muscle strength and biomarkers

**Fig. 2:**
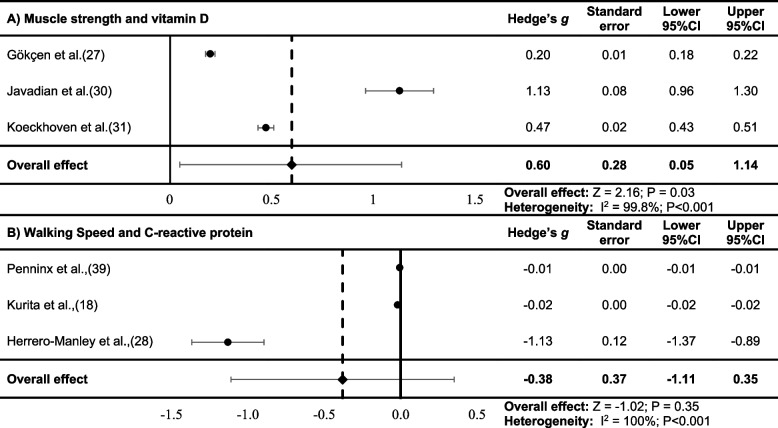
Forest plot for the random-effect meta-analysis for muscle strength and vitamin D (**A**), walking speed and C-reactive protein (**B**) 95%CI; 95% confidence interval

Thirteen studies reported lower limb muscle strength including peak isometric force [23], isokinetic knee flexor and extensor torque [27, 42]. Meta-analyses revealed that lower limb muscle strength and vitamin D were significantly associated (Hedge’s *g:* 0.60; Lower 95%CI: 0.05; Upper 95%CI: 1.14 SE: 0.28; *P* = 0.03), see Fig. 2a. [27, 30, 31]. No evidence of publication bias was evident, although there was significant heterogeneity (I^2^ = 99.8%; *P* < 0.001). Across all available studies, associations between lower limb skeletal muscle strength and biomarkers were largely focused on inflammatory markers, with significant associations between muscle strength and biomarkers of oxidative stress (Table 4). No significant associations were reported between lower limb skeletal muscle strength measures and other measures of inflammation, cardiometabolic or genetic biomarkers (Table 4).

**Table 4:** Study statistical analysis and results from papers examining the relationship between muscle measure and biomarkers

Author	OA sample size	Statistical analysis	Outcomes
Barker, et al. [23]	29	Pearson Product Moment Linear correlation	Markers of oxidative stress (Cu/Zn SOD, Mn SOD) were significantly associated (0.38 > *r* < 0.47, *P* < 0.05) with muscle strength (force, torque, knee flexion and extension). There was no association (0.0 > *r* < 0.34, *P* > 0.05) between markers of inflammation (IL-1β, IL-2, IL-4, IL-5, IL-6, IL-7, IL-8, IL-10, IL-11, IL-12, IL-13, GM-CSF, IFN-γ, TNF-α, IL-1r1, IL-1r2, IL1-4r, IL-6r, sTNFr-1, sTNFr-2) and muscle strength
*USA*			
Chang, et al. [24]	100 Elderly (> 65y) = 74 Middle-aged (40-64y) = 26	Pearson’s Product Moment Linear correlation	Coenzyme Q10/TC (umol/mmol) was significantly associated with leg-back strength (kg) in elderly adults (> 65 years) with OA (r = 0.29; *P* < 0.05) and gait speed (m/s; r = 0.33; *P* < 0.01). Yet, there was no significant association to chair-stand test (reps). There was no significant relationship between coenzyme Q10/TC (umol/mmol) and muscle assessment in middle-aged adults (40–64 years)
*Taiwan*		Spearman’s rank order correlation	
Durmus, et al. [11]	37	Spearman’s rank order correlation	No association was seen between leptin levels and muscle strength (right *r* = 0.72, *P* = 0.06, left *r* = 0.15, *P* = 0.35) or 6-min walk distance (*r* = -0.20, *P* = 0.24)
*Turkey*			
El-Fetiany, et al. [25]	90	Pearson’s Product Moment Linear correlation	No significant correlation was found between plasma bFGF levels and 6MWT, stair climb test, or chair stand test. [No data were presented]
*Egypt*			
Glover, et al. [26]*	256	Pearson’s correlations	Vitamin D was associated with short physical performance battery (*r* = -0.20, *P* < 0.01)
*USA*			
Gökçen, et al. [27]	152	Spearman’s rank order correlation	No association was seen between muscle strength (Isometric, Isokinetic for knee extension and flexion, and manual muscle test) and Osteocalcin (0.01 > *r* < 0.16), CTX-1 (0.05 > *r* < 0.14), CTX-II (0.0 > *r* < 0.18), leptin (0.16 > *r* < 0.16), and 25(OH)D (0.03 > *r* < 0.10)
*Turkey*			
Herrero-Manley, et al. [28]	48	Pearson’s Product Moment Linear correlation	Uric acid and CRP was associated with walking speed (*r* = -0.48, *P* < 0.05; *r* = 0.34, *P* < 0.05) and sit-to-stand (*r* = -0.39, *P* < 0.05; *r* = -0.38, *P* < 0.05). There was no association between walking total cholesterol, HDL, LDL and triglycerides with walking speed and sit-to-stand (r range 0.004 to 0.20, *P* > 0.05)
*Spain*			
Hunt et al	17	Linear regression model	KAM impulse predicted significant variation in urinary CTX-II (β = 1.19, 95%CI = 0.16, 2.21; *p* = 0.05) and uCTX-II: serum CPII ratio (β = 1.50, 9%%CI = 0.72, 2.28, *P* < 0.01) when K&L and walking speed were added to the regression models CTX-II was no longer significant. Peak KAM, and KAM impulse were no associated with urinary C2C, serum HA, serum COMP, or urine C2C:serumCPII ratio or serum HA:serum CPII ratio. Associations between muscle strength and biomarkers were not reported
*Canada*			
Javadian, et al. [30]	92	Pearson’s Product Moment Linear correlation	Quadriceps muscle strength correlated positively with 25(OD)D (*r* = 0.304, *P* = 0.005), after adjustment for age, sex, and body mass index (*r* = 0.496, *P* = 0.01)
*Iran*		Linear regression analysis	
Koeckhoven et al. [31]	319	Univariable linear regression Multivariable linear regression	25(OH)D was significantly associated with muscle strength (β = 0.204, 95%CI = 0.014, 0.050), *P* < 0.001, 319) and remained significant after adjustment for season of blood collection, alcohol consumption, number of comorbidities and sex (β = 0.181, 95%CI = 0.014, 0.043), *P* < 0.001)
*Netherlands*			
Kurita, et al. [18]	1425	Logistic regression model	Serum CK (mean difference 0.02, 95%CI = 0.01, 0.03, *P* < 0.001), but not CRP (mean difference 0.02, 95%CI = 0.01, 0.03, *P* < 0.001), ALT (mean difference 0.02, 95%CI = 0.01, 0.03, *P* < 0.001) or AST (mean difference 0.02, 95%CI = 0.01, 0.03, *P* < 0.001) were associated with ASMI. CRP (mean difference -0.02, 95%CI = -0.03, -0.01, *P* < 0.001) and ALT (mean difference 0.01, 95%CI = 0.00, 0.02, *P* = 0.049) but not CK (mean difference 0.00, 95%CI = 0.00, 0.00, *P* = 0.896) and AST (mean difference 0.01, 95%CI = 0.00, 0.03, *P* = 0.088) were associated with gait speed
*Japan*			
Levinger, et al. [32]	33	Spearman’s rank order correlation	Muscle strength was significantly negatively associated with MCP-1 (*r* = -0.37, *P* = 0.042) and gene expression of TNF-α (*r* = -0.46, *P* = 0.012), and atrogin-1 mRNA (*r* = -0.36, *P* = 0.04). No associations between muscle strength and SOCS-3 mRNA, total cellular protein of inflammatory kinases (STAT3, JNK2, JNK1, NF-kB p65), IL-8, IL-6) were reported
*Australia*			
Levinger, et al. [33]	19	Pearson’s Product Moment Linear correlation	Total cellular protein of inflammatory kinases (NF-kB p65, STAT3) were negatively associated with gait velocity (*r* = -0.52, *P* = 0.016; *r* = -0.46, *P* = 0.032 respectively), MCP-1, JNK1, or IL-6 were not associated with gait velocity (r range -0.29 to 0.23, *P* > 0.05). Knee sagittal impulse was negatively associated with JNK-1 and MCP-1 (*r* = -0.49, *P* = 0.01; r-0.52, *P* = 0.023 respectively). No other associations were seen
*Australia*			
Levinger, et al. [34]	19	Spearman’s rank order correlation	Reduced muscle strength was associated with higher levels of FoxO1 expression in the muscles (*r* = -0.56, *P* = 0.03). No associations with IL-15 were reported
*Australia*			
Manoy, et al. [35]	208	Pearson’s Product Moment Linear correlation Multivariable linear regression	Serum leptin demonstrated a weak association with physical performance; gait speed (*ρ* = -0.25, *P* < 0.001), TUG (*ρ* = 0.27, *P* < 0.001), STS (*ρ* = 0.27, *P* < 0.001) and 6MWT (*ρ* = -0.24, *P* < 0.001). There was no association between serum leptin levels and ASMI (*ρ* = 0.08). In multivariable regression adjusted for age, sex, BMI and fat mass, serum leptin levels were associated with knee extension force (*r* = -0.119, *P* = 0.039) and 6MWT (*r* = -0.139, *P* = 0.029)
*Thailand*			
Manoy, et al. [36]	262	Spearman's Rank Correlation	Blood leukocyte RTL weak association with gait speed (*ρ* = 0.20, *P* = 0.004), 6MWT (*ρ* = 0.21, *P* = 0.003), TUG (*ρ* = 0.16, *P* = 0.03), and STS (*ρ* = 0.15, *P* = 0.03) and no association with knee extensor force (*ρ* = 0.02, *P* = 0.77). Multivariate linear demonstrated blood leukocytes RTL was associated with gait speed (β = 0.185 (95%CI 0.031, 0.407), *P* = 0.023), TUG (β = -0.189 (95%CI -0.032, 0.002), *P* = 0.025), STS (β = -0.231 (95%CI -0.019, 0.004), *P* = 0.004), and 6MWT (β = 0.191 (95%CI 0.000, 0.001), *P* = 0.022), but not knee extensor force (β = 0.004 (95%CI -0.008, 0.008), *P* = 0.948)
*Thailand*		Multivariable linear regression	
Miller, et al. [37]	87	Linear regression analysis	Inflammatory markers of soluble receptors for tumour necrosis factor-alpha (TNF-α; sTNFR1, sTNFR2) were associated with stair climb time (β = 0.389, *P* = 0.003; β = 0.317, *P* = 0.02) when adjusted for age, sex, race, BMI, comorbid conditions, and NSAID use. CRP was associated with distance walked (β = -0.324, *P* = 0.08) in unadjusted analysis but was no longer associated in adjusted analysis. IL-6 and TNF-α were not associated with stair climb time or 6MWT distance
*USA*			
Miller, et al. [38]	309	Spearman’s rank order correlation	Distance walked was associated with SHBG (*r* = -0.33, *P* = 0.01, *n* = 70) in men only, no associations were seen between distance walked and cortisol (men *r* = 0.09, *P* = 0.45, *n* = 70; women *r* = -0.11, *P* = 0.14, *n* = 168), DHEA (men *r* = -0.07, *P* = 0.63, *n* = 57; women *r* = 0.13, *P* = 0.22, *n* = 89), growth hormone (men *r* = -0.03, *P* = 0.85, *n* = 50; women *r* = -0.13, *P* = 0.11, *n* = 151), T-testosterone (men *r* = 0.04, *P* = 0.74, *n* = 70; women *r* = -0.08, *P* = 0.45, *n* = 81), and SHBG in women (= -0.05, *P* = 0.51, *n* = 158)
*USA*			
Pagura, et al. [12]	139	Pearson’s Product Moment Linear correlation	Non-significant poor to moderate associations were reported between IGF-1 and percentage lean body mass, fast self-paced walk, normal self-paced walk, TUG and stair negotiation (no data presented)
*USA*		Multivariable linear regression	
Penninx, et al. [39]	274	Spearman’s rank order correlation	Walking speed was associated with IL-6 (β = -0.036, *P* = 0.08) when adjusted for age, sex, race, BMI, coronary heart disease, congestive heart failure, diabetes, cancer, lung disease, NSAID use. No association was seen between walking speed and CRP (β = -0.008, *P* = 0.37), TNF-α (β = 0.021, *P* = 0.13) and IL-6r (β = -0.0003, *P* = 0.78) IL-1r (β = -0.001, *P* = 0.74), and sTNFR1 (β = -0.052, *P* = 0.22) and sTNFR2 (β = -0.001, *P* = 0.20)
*USA*		Multivariable linear regression	
Sakr, et al. [40]	82 Egyptians = 41 Yemeni = 41)	Spearman’s rank order correlation	25(OH)D was not associated with the 6MWT or chair stand test in Egyptians (*r* = 0.2, *P* = 0.2; *r* = 0.1, *P* = 0.2) and Yemeni (*r* = 0.03, *P* = 0.3; *r* = 0.1, *P* = 0.2) participants
*Egypt*		Multivariate logistic regression	
Sanchez-Ramirez, et al. [41]	285	Multivariable linear regression	Muscle strength was associated with inflammatory markers of CRP (β = -0.13, *P* = 0.03) and ESR (β = -0.21, *P* < 0.001) when adjusted for age, gender, comorbidities, NSAID use and BMI were no longer associated (CRP β = 0.04, *P* = 0.44; ESR β = 0.02, *P* = 0.67)
*Netherlands*			
Santos, et al. [42]	80	Spearman’s rank order correlation	Plasma IL-6 was associated with muscular balance (hamstrings: quadriceps ratio r = 0.254, *P* = 0.023) and peak torque/body mass of the knee flexors (*r* = -0.232, *P* = 0.03), but not extensors (data not reported)
*Brazil*			
Selistre, et al. [43]	25	Pearson’s Product Moment Linear correlation	A significant correlation was observed between urinary CTX-II amd 40 m walk test (*r* = -0.48, *P* = 0.04) and gait speed (*r* = -0.54, *P* = 0.03) but not peak KAM (*r* = -0.04, *P* = 0.89), peak knee flexion moment (*r* = 0.03, *P* = 0.55) or knee adduction angular impulse (*r* = 0.14, *P* = 0.90). After controlling for severity and BMI, urinary CTX-II explained an additional 7% of variance to severity and BMI (*R* = 0.68, *R*^2^ = 0.46, change in *R*^2^ = 0.07, *P* = 0.03)
*Brazil*		Hierarchical linear regression	
Udomsinprasert, et al. [44]	175	Pearson’s Product Moment Linear correlation	Serum adiponectin levels were associated with ASMI (*r* = -0.22, *P* = 0.003), skeletal muscle mass index (*r* = 0.43, *P* < 0.001), gait speed (*r* = -0.36, *P* < 0.001), TUG (*r* = -0.27, *P* < 0.001), STS (*r* = -0.21, *P* = 0.007), and 6MWT (*r* = 0.37, *P* < 0.001), but not knee extensor strength (*r* = 0.009, *P* = 0.231)
*Thailand*		Multivariable linear regression	

^*^Excluded due to high risk of bias

*25(OH)D* vitamin D, *6MWT* 6-min walk test, *ALT* Alanine aminotransferase, *ASMI* Appendicular Skeletal Muscle Index, *AST* Aspartate aminotransferase, *bFGF* Basic fibroblast growth factor, *BMI* Body Mass Index, *C2C* Cleavage of type ii collagen by collagenases, *CK* Creatine phosphokinase, *CPII* Type II Procollagen C-Propeptide, *COMP* Cartilage oligomeric matrix protein, *CRP* C-reactive protein, *CTX-I* C-terminal telopeptide type I collagen, *CTX-II* C-terminal telopeptide type II collagen, *Cu/ZnSOD* Cu/Zn Superoxide Dismutase, *DHEA* Dehydroepiandrosterone, *ESR* Erythrocyte sedimentation rate, *Fox O1* Forkhead box protein O1, *HA* Hyaluronic acid, *HDL* High-density lipoprotein, *IFN-γ* Interferon-gamma, *IGF-1* Insulin-like growth factor-1, *IL-1β* Interleukin 1 beta, *IL-1r* Interleukin 1 receptor, *IL-1r1* Interleukin 1 receptor 1, *IL-1r2* Interleukin 1 receptor 2, *IL-10* Interleukin 10, *IL-12* Interleukin 12, *IL-13* Interleukin 13, *IL-15* Interleukin 15, *IL-17* Interleukin 17, *IL-18* Interleukin 187, *IL-2* Interleukin 2, *IL-4* Interleukin 4, *IL-4r* Interleukin 4 receptor, *IL-5* Interleukin 5, *IL-6* Interleukin 6, *IL-6r* Interleukin 6 receptor, *IL-7* Interleukin 7, *IL-8* Interleukin 8, *JNK* c-Jun N-terminal kinase, *KAM* Knee adduction moment, *LDL* Low-density lipoprotein, *MCP-1* Monocyte Chemoattractant Protein-1, *Mn SOD* Manganese Superoxide Dismutase, *NSAID* Nonsteroidal anti-inflammatory drugs, *Q10* Ubiquinone-10, *RNA* Ribonucleic acid, *RTL* Relative Telomere Length, *SHBG* Sex Hormone Binding Globulin, *SOCS* Suppressor of cytokine signalling 3, *STAT-3* Signal Transducer and Activator of Transcription 3, *STS* Sit-to-stand, *sTNFR1* Soluble forms tumour necrosis factor alpha receptor 1, *sTNFR2* Soluble forms tumour necrosis factor alpha receptor 2, *TC* Tri-circulator, *TNF-α* Tumour necrosis factor alpha, *TUG* Timed-up and go

### Walking speed and biomarkers

Walking speed was collected from a variety of testing measures, including the 6-min walk test (6MWT) [24, 25, 36, 38–40, 44, 50], 10-m walk test [18, 28], 40-m walk test [43], and self-paced walking [12, 33]; data displayed in Table 3. Reduced walking speed was unfavourably non-significantly associated with c-reactive protein (CRP) (Hedge’s *g*: -0.38; SE: 0.37; Lower 95%CI: -1.11; Upper 95%CI: 0.35; *P* = 0.35) [18, 28, 39], see Fig. 2b. No evidence of publication bias was evident, although there was significant heterogeneity (I^2^ = 100%; *P* < 0.001).

A total of 41 biomarkers including inflammatory (e.g., TNF-α) [39], energy metabolism (e.g., high- and low- density lipoprotein) [28], and hormone markers (e.g., dehydroepiandrosterone sulphate (DHEA)) [38] were examined with walking speed. There were significant associations between walking speed and biomarkers primary characterised with oxidative stress (coenzyme Ubiquinone-10 (Q10) [24], coenzyme Q10/Tri-circulator [24]), inflammation (Nuclear Factor-kB p65 [33], Signal Transducer and Activator of Transcription 3 (STAT-3) [33], soluble forms tumour necrosis factor alpha receptor 2 (sTNFR2) [37]), vitamin D [40], enzyme (Alanine aminotransferase [18]), metabolic (blood leukocyte relative telomere length [36]), hormone (serum leptin [35]), glycoprotein (sex hormone-binding globulin (SHBG) [38]), and bone urinary uCTX-II [43]) or inflammation (Interleukin 1 receptor (IL-1r) [39], Interleukin 6 (IL-6) [33, 39], IL-6 174 G/C [50], Interleukin 6 receptor [39], monocyte Chemoattractant Protein-1 (MCP-1) [33], Nuclear Factor-kB p65 [33], TNF-α [39], TNF-α 238 G/A [50], TNF-α 308 G/A [50], Soluble forms tumour necrosis factor alpha receptor 1 (sTNFR1) + 36 A/G [50], sTNFR2 + 1663 A/G [50], sTNFR2 + 676 T/G [50], sTNFR1 [37, 39], sTNFR2 [39]), hormones (DHEA [38], growth hormone [38], testosterone [38]), stress (cortisol [38], c-Jun N-terminal kinases-1 [33]), metabolic (basic fibroblast growth factor [25], creatine kinase [18]) and enzymes (aspartate transaminase [18]) (Table 4).

### Functional assessment and biomarkers

Lower limb muscle function was predominantly assessed using chair sit to stand [24, 25, 28, 35, 36, 40, 44], get-up and go [35, 36, 44, 49], or climbing stairs [12, 37]. Studies included a combination of functional tests using the Short Physical Performance Battery [24, 26], or used four tests to determine ‘Physical Performance’ (4-m gait speed test, get-up and go, five times sit-to-stand tests, and 6MWT) [47]. Biomarkers associated with functional assessment measures included energy metabolism (e.g., cholesterol, high- and low- density lipoprotein, and triglycerides) [28], inflammatory markers (e.g., sTNFR1and sTNFR2 [37, 50], CRP [28]), vitamin markers (e.g., vitamin D) [26, 40, 47, 49], and hormone markers (e.g., leptin) [35].

## Discussion

The current study summarised existing literature exploring the relationship between biomarkers and lower limb skeletal muscle dysfunction in adults with OA. Numerous studies reported associations between biomarkers and lower limb skeletal muscle measures, with a lack of consistency in both biomarkers and lower limb skeletal muscle measures, and limited muscle -specific markers. Our meta-analysis identified lower limb skeletal muscle strength was significantly associated with vitamin D (Hedge’s *g*: 0.60; *P* = 0.03), however, walking speed, an indicator of muscle function, was not significantly associated with CRP (Hedge’s *g*: -0.38; *P* = 0.35). Both meta-analyses displayed no publication bias based on visual inspection of the funnel plots, yet there was significant heterogeneity. It is evident from this review that there is a growing breadth, but not depth, of research in this area, making it difficult to synthesise and draw clear conclusions. Therefore, the relationship between biomarkers and lower limb skeletal muscle dysfunction in adults with OA remains unclear.

Evidently, research in the area is evolving with 93 biomarkers identified in this review, predominantly characterised as inflammatory (*n* = 35), metabolic (*n* = 15) and hormone (*n* = 10). The high level of interest in inflammatory and metabolic markers is unsurprising given their link to distinct OA phenotypes [6]. Inflammation is associated with protein abundance, linked with muscle strength and atrophy [51]. With emerging evidence of the role of inflammation in OA [52] clarifying which markers are involved in different aspects of the disease process is important. Whilst metabolic alterations have been specifically linked to bone and cartilage [6], various metabolites may also directly contribute to inflammation [53]. Due to a lack of studies, only one meta-analysis was undertaken using inflammatory markers (CRP). Four of the 15 metabolite markers identified demonstrated associations with lower limb skeletal muscle dysfunction [18, 28, 33, 36]. Biomarkers such as creatine phosphokinase, and uric acid may have a specific muscle role such as cell breakdown and muscle disturbance, whilst markers such as Forkhead box protein O1 (FoxO1) and blood leukocyte telomere length, may have either dual roles or act through other channels. It is, therefore, important to identify biomarkers associated with skeletal muscle dysfunction and understand the mechanistic association.

Associations between a growing number of potential biomarkers were identified. Surprisingly, there were limited muscle- specific markers reported, likely due to very few studies exploring muscle-specific biomarkers [23]. Most studies explored generic biomarkers with lower limb muscle measures as a secondary outcome. Six clinical phenotypes and nine endotypes of knee OA have been identified, with it likely that the future biomarkers of prognosis or efficacy of a treatment will be part of these molecular pathways [54]. Many biomarkers identified within the current review are classified as cartilage-driven, metabolic, bone, and synovitis-driven phenotypes. The OA-specific markers identified were mainly cartilage- (CTX-II, C2C) and synovitis-driven (TNF-α, IL-1), linked with cartilage degradation and high levels of systemic inflammation [54]. Systemic inflammation may trigger protein catabolism and impair the anabolic response whereby an increase in proinflammatory cytokines (e.g., TNF-α, IL-1, IL-6) is associated with muscular atrophy [55]. Furthermore, muscular dysfunction may accelerate the inflammatory process, leading to the exacerbation of cartilage degradation [56]. Key energy metabolites such as adenosine triphosphate (ATP) and glucose, are fundamental to muscle contraction [57]. These same metabolites are upregulated to maintain and repair cartilage [58], highlighting the role of metabolites in the OA disease progress. That said, direct and indirect pathways through which metabolites are associated with both muscle and OA, and how these two pathways coincide remains unclear. Understanding these metabolic pathways, could aid in the understanding of early diagnosis, management of OA and prevention of OA-related disability. For biomarkers to be true measures of OA muscle dysfunction, they need to be associated with measures of OA and muscle or demonstrate differences in the associations between OA and controls. Of the 24 papers, only 13 (50%) reported either differences between OA and controls for the biomarkers and/or muscle measures (*n* = 9) or reported associations between biomarkers and OA (*n* = 6). Interestingly, Gocken and colleagues [27] reported that vitamin D did not differ across K&L grade. Whilst this doesn’t preclude differences between OA and controls, the other studies also only included individuals with OA which precludes a comparison. Given the lack of available information, currently we are unable to confirm which biomarkers are associated with muscle dysfunction in OA.

Vitamin D research has expanded rapidly in the last 10 years, in part due to the high prevalence of vitamin D deficiency in OA [59]. Vitamin D signalling plays an important part in adipose tissue [60]. Changes in muscle properties including intermuscular adiposity gains, seen in OA [61], may explain the link between muscle properties, which influences lower limb muscle strength and vitamin D. Given this larger body of evidence exploring the role of vitamin D, cross-sectional data was only available to examine the relationship with muscle strength.

One of the key considerations highlighted by this review is the high level of heterogeneity evident. There are several factors which could have led to this. Whilst all studies included in the current article assessed knee OA using radiographic and ACR criteria, muscle strength was assessed differently using isokinetic muscle contractions at 90 degrees/second, or Isotonic contractions [31]. Of the studies included in this review, those with larger samples reported no association [16, 37], used radiographic criteria for inclusion and reported combined hip and knee OA [16]. There is also large variation in participants included within this review, different OA characteristics and treatment approaches would be in place and thus might influence any reported outcomes. There may also be key environmental conditions and external influences that may have impacted these individuals. Unfortunately, additional analysis to explore heterogeneity couldn’t be undertaken due to the few included studies, yet these factors may explain some of the variance between studies.

It is valuable to consider the multifactorial nature of OA [62], and the distinct phenotypes identified. There may be single biomarkers of interest relevant to some phenotypes, such as inflammatory markers linked also linked with the inflammatory phenotype, however a composition of multiple biomarkers (biomarker signatures) from multiple mechanistic pathways may provide greater insight [63]. The wide range of the biomarkers indicates an evolving research field, yet there remains a lack of replication and confirmation, with wide-ranging assessments of lower limb skeletal muscle dysfunction. Future research must consider the validation and confirmation of biomarkers and association with muscle dysfunction. The biomarkers identified were circulating systematic markers derived from blood or urine, only one study explored markers from muscle biopsies [34]. Circulating systemic markers of skeletal muscle assume the biomarkers have been secreted from the skeletal muscles [64]. This assumption may hold if the study’s primary aim was to assess biomarkers of skeletal muscle, however this was not always the case. Some potential markers may have therefore been overlooked, whilst others that are included in this review may not be related. Blood and urine samples are frequently reported, likely due to factors such as being more feasible and less invasive, compared to direct muscle assessment measures. Circulatory markers may be more clinically relevant, yet mechanistically, identification of markers from skeletal muscle specimens is required to fully understand skeletal muscle changes. As such, this may in turn explain the biomarkers secreted and circulated.

Lower limb skeletal muscle dysfunction is assessed in various ways (e.g., manual muscle tests, isokinetic contractions, isotonic contractions). Skeletal muscle strength plays a large role in mobility-related disability and skeletal muscle dysfunction, such as muscle activation and tissue attenuation [65]. Muscle dysfunction is not the sole driver of disability. Pain and stiffness play a role in making daily activities uncomfortable and difficult, resulting in avoidance behaviours [66]. However, pain and stiffness also influence muscle dysfunction, having been linked to atherogenic muscle inhibition the inability to fully activate the muscles due to atrophy and neural inhibition [67]. Understanding the interplay between muscle dysfunction and joint health (e.g., pain, stiffness, function) is crucial for improving mobility quality.

There are currently no recommendations for assessing skeletal muscle in individuals with OA, and 45% of individuals with OA also have sarcopenia [68], assessments for sarcopenia rely predominantly on muscle mass and handgrip strength, depending on the classification criteria used [69–71]. The current review focused on lower limb muscle dysfunction due to its links with mobility-related disability, however when explored further, it will be important to understand the link between systemic circulating markers and skeletal dysfunction at sites distant to the site of OA (e.g., knee OA, with upper limb strength). When exploring biomarkers of lower limb skeletal muscle dysfunction in OA and comparing them to our previous work in sarcopenia [72], some markers (e.g., interleukins) overlap, while others may be condition-specific or yet to be explored in the other condition. Markers of sarcopenia may also have some relevance to OA, given the prevalence of sarcopenia in individuals with OA.

There were also inconsistencies in defining OA, 24 studies included knee OA, one included both hip and knee OA [18]. Whilst hip and knee OA demonstrate similar muscle dysfunction patterns [17, 18], they also have different etiologies. There was a lack of studies exploring hip OA preventing sensitivity analysis. Future work needs to explore the relationships in hip OA or be adequately powered to conduct analysis by joint. There was also a large variation in the definition of OA, from radiographic K&L grades, ACR criteria, and joint replacement waiting lists. Furthermore K&L grades varied from early OA (0–1) [28] to moderate and severe OA (2–4) [40]. These variances may account for inconsistencies in the associations between biomarkers and lower limb skeletal muscle dysfunction. Only one study [28] defined early OA, others used different OA criteria (e.g., radiographic, ACR) and thresholds. None of the studies explored the influence of disease severity on the association. Most studies defined disease severity based on radiographic evidence. Whilst muscle weakness is a risk factor for OA [73], symptomatic OA progression has been associated with greater muscle weakness, atrophy and loss of muscle specific strength, whereas radiographic severity has been associated with greater intramuscular fat [61, 74]. Given the different skeletal muscle dysfunction patterns with OA progression studies should not only consider disease stage but also radiographic and symptomatic progression when identifying biomarkers.

Given that OA is more prevalent in females [75], it is unsurprising that 78% of participants included were female. Two studies were single sex, the remaining were mixed sex, only one study [36] stratified by sex. Fewer than 50% of included studies accounted for sex in the analysis. That said, there is an abundance of literature demonstrating differences in skeletal muscle function between sexes. Females with OA demonstrate higher muscle co-activation [76], increased intra-muscular fat, reduced fibre tissue [77] other differences include strength, muscle morphology, and mobility [78–80]. Further research is required to understand sex-specific pathophysiology mechanisms for OA, and/or account for sex in the analysis.

The current review evaluated study quality using the Joanna Briggs Institute Checklist for analytical cross-sectional studies. Several studies lacked appropriate statistical information (Table 2), sometimes impacting the quality of analysis and data provided. One paper was excluded [26], and the corresponding authors for four papers were contacted for further information, however, they failed to respond. Greater transparency, and data and information sharing along with the examination of confounding variables, assessments of multiple relationships within set models, the inclusion of confidence intervals and following reporting guidelines such as EQUATOR are required [81, 82].

Comorbidities are prevalent in 67% of individuals with OA [83]. Individuals included in the study likely had comorbidities; however, this was unable to be accounted for in the analysis. Understanding of the relationship between biomarkers and lower limb skeletal muscle dysfunction in OA, especially given the influence comorbidities can have on both biomarkers, and lower limb skeletal muscle function, is important. Given the requirements for real-world knowledge and recommendations, confirmation of associations between biomarkers and lower limb skeletal muscle measures are required in individuals with and without comorbidities. Although not an easy task, future research may need to consider many influencing factors such as time since diagnosis, severity, therapeutics etc., which could significantly influence the associations, thus endeavouring to unpick this complex and multifactorial relationship.

## Conclusions

In conclusion, a lack of replication of biomarkers and heterogeneity of these biomarkers and lower limb muscle measures makes understanding this relationship difficult, and results should be interpreted with caution. Associations between variables was limited, the few studies exploring lower limb muscle whereby measures were mainly secondary outcomes. There was a wide range of predominantly generic biomarkers related to overall health, with a lack of muscle- and osteoarthritis-specific biomarkers. As such, the mechanistic pathways through which these associations occur are less evident, and difficult to draw clear conclusions on these relationships. Future research needs to focus on muscle specific markers including exploring molecular changes beyond generic markers such as histological changes, markers from muscle specimens and markers likely excreted from the muscle. Furthermore, understanding the pathophysiological mechanisms will enable a greater understanding of markers likely identify changes preceding functional decline.

## Supplementary Information

Supplementary Material 1.

## Data Availability

No datasets were generated or analysed during the current study.

## Abbreviations

6MWT: 6-Minute walk test
ACR: American College of Rheumatology
C2C: Cleavage of type ii collagen by collagenases
COMP: Cartilage oligomeric matrix protein
CTX-II: C-terminal telopeptide type II collagen
DHEA: Dehydroepiandrosterone sulphate
IGF-1: Insulin-like growth factor-1
IL-1: Interleukin 1
IL-1r: Interleukin 1 receptor
IL-6: Interleukin 6
K&L: Kellgren and Lawrence grade
MCP-1: Monocyte Chemoattractant Protein-1
OA: Osteoarthritis
PICOS: Population, Intervention, Comparator, Outcomes and Study design
PRISMA: Preferred Reporting Items for Systematic Reviews and Meta-Analyses
Q10: Ubiquinone-10
SD: Standard deviation
SE: Standard error
SHBG: Sex hormone-binding globulin
SMD: Standardised mean difference
STAT-3: Signal Transducer and Activator of Transcription 3
sTNFR2: Soluble forms tumour necrosis factor alpha receptor 2
TNF-a: Tumour necrosis factor alpha
