# Supplementary material for: Associations between biomarkers and skeletal muscle function in individuals with osteoarthritis: a systematic review and meta-analysis
Source: Arthritis Res Ther. 2024 Nov 5;26:189. doi: 10.1186/s13075-024-03419-1 (PMC11536556; doi:10.1186/s13075-024-03419-1)
Supplement: Supplementary file 1 — Supplementary Material 1. [file 13075_2024_3419_MOESM1_ESM.docx]

**Supplementary Materials**

| **Supplementary Table 1.** Study PubMed search strategy | |
| --- | --- |
| #1. | (((((biomarkers[MeSH Terms]) OR (biomarkers)) OR (biomark*)) OR (marker*)) OR (biological marker*)) OR (biological signature*) |
| #2. | ((((metabolite marker*) OR (metabolomics)) OR (metabolism)) OR (metabolome)) OR (metabolomics[MeSH Terms]) |
| #3. | ((((((((proteomic[MeSH Terms]) OR (genomics[MeSH Terms])) OR (transcriptomics)) OR (proteomics)) OR (genomics)) OR (transcriptome)) OR (proteome)) OR (genome)) OR (omics) |
| #4. | Molecular |
| #5. | ((histology[MeSH Terms]) OR (histology)) OR (histological) |
| #6. | (physiological) OR (physiology) |
| #7. | (((((inflammation[MeSH Terms]) OR (inflam*)) OR (proinflam)) OR (metabo*)) OR (cytokin*)) OR (interleukin*)) OR (chemokin*) |
| #8. | **#1 OR #2 OR #3 OR #4 OR #5 OR #6 OR #7** |
| #9. | osteoarthritis[MeSH Terms] OR osteoarthritides[MeSH Terms] OR osteoarthritis OR osteoa* OR osteoarthrosis OR polyosteoarthritis OR gonarthrosis OR OA OR KOA OR HOA |
| #10. | (((lower extremity[MeSH Terms]) OR (lower extremities[MeSH Terms])) OR (lower extremit*)) OR (lower extremity) |
| #11. | (knee) OR (knees) |
| #12. | (leg) or (legs) |
| #13. | (hip) OR (hips) |
| #14. | ankle* |
| #15. | (foot) OR (feet) |
| #16. | (ankle) OR (ankles) |
| #17. | (thigh) OR (shank) |
| #18. | (lower limb) OR (lower limbs) |
| #19. | **#10 OR #11 OR #12 OR #13 OR #14 OR #15 OR #16 OR #17 OR #18** |
| #20. | (muscle) OR (muscle*) |
| #21. | Strength |
| #22. | Function |
| #23. | Quality |
| #24. | ((((activation) OR (co-activation)) OR (coactivation)) OR (cocontraction)) OR (co-contraction) |
| #25. | (((cross-sectional area) OR (anatomical cross-sectional area)) OR (CSA)) OR (ACSA) |
| #26. | (intramuscular fat) OR (intra-muscular fat) |
| #27. | Gait OR Timed-up-and-go OR timed up and go OR sit-to-stand OR sit to stand OR STS OR TUG OR stairs |
| #28. | **#20 OR #21 OR #22 OR #23 OR #24 OR #25 OR #26 OR #27** |
| #29. | **#8 AND #9 AND #19 AND #28** |

| **Supplementary Table 2:** Study characteristics of papers, including lower limb muscle measure and a biological marker, not reporting correlations. | | | | | | | | | | | |
| --- | --- | --- | --- | --- | --- | --- | --- | --- | --- | --- | --- |
| **Author** | **Study Design** | **Total**  **Sample**  **Size** | **OA subgroup** | **OA**  **location** | **OA subgroup sample size** | **Age (years)** | **Number of females (%)** | **K&L Grade** | **ACR** | **Lower limb muscle measure** | **Biological marker** |
| Aguiar, Do Nascimento (1) | INV | 22 | Whole group | Knee | 22 | 59 ± 6 | 18 (82) | 3 (median) | No | Thigh circumference, training load, rang of motion | IL-6, TNF-α, sTNFR1, sTNFR2 |
| *Brazil* |  |  |  |  |  |  |  |  |  |  |  |
| Apparao, S (2) | OBS | 20 | Whole group | Knee | 20 | 36 to 65 | N/A | 1 and 2 | Yes | Handheld dynamometer, knee extensors and flexion strength | sCOMP |
| *Chennai, India* |  |  |  |  |  |  |  |  |  |  |  |
| Armagan, Serin (3) | RCT | 50 | Group 1 | Knee | 30 | 63 ± 6 | 30 (100) | 2 and 3 | Yes | 20 m walking time | CTX-II, IL-1β, MMP-3 levels, Nitric oxide |
| *Turkey* |  |  | Group 2 | Knee | 20 | 60 ± 7 | 20 (100) | 2 and 3 | Yes |  |  |
| Arden, Cro (4) | RCT | 474 | Vitamin D | Knee | 237 | 64 ± 8 | 144 (61) | 0 to 4 | No | Get-up and go, muscle strength, handgrip strength, Time to walk 10m | 20(OH)D3 |
| *UK* |  |  | Placebo | Knee | 237 | 64 ± 8 | 145 (61) | 0 to 4 | No |  |  |
| Azukizawa, Ito (5) | INV | 42 | Whole group | Knee | 42 | 59 ± 6 | 42 (100) | 0/1 (n =32)  2/3 (n=10) | No | 3-minute walk, 30 sec sit-to-stand, timed up and go | sCOMP, sPiiCP, uC2C, uCTX-II |
| *Japan* |  |  |  |  |  |  |  |  |  |  |  |
| Barker, Henriksen (6) | OBS | 56 | Vitamin D deficient | Knee | 17 | 51 (3) | 8 (47) | >2 | No | Symptomatic acceleration knee extension, flexion, average power knee flexion, deceleration knee flexion, speak torque knee flexion, total work knee flexion, | 25(OH)D, Ascorbic acid, Calcium, GM-CSF, Hs-CRP, IFN-γ, IL-1 β, IL-10, IL-12, IL-13, IL-2, IL-4, IL-5, IL-6, IL-7, IL-8, iPTH, Lutein, Rheumatoid factor, TNF-α, Uric acid, Zeaxanthin, α-Carotene, α-Tocopherol, β-Carotene, |
| *USA* |  |  | Vitamin D insufficient | Knee | 21 | 48 (2) | 12 (57) | >2 | No |  |  |
|  |  |  | Vitamin D sufficient | Knee | 18 | 47 (3) | 11 (61) | >2 | No |  |  |
| Bischoff-Ferrari, Orav (7) | RCT | 273 | Whole group | Knee | 273 | 70 ± 6 | 146 (54) | 0 to 4 | No | Gait speed 4 m, repeated sit-to-stand test | 25(OH)D, iPTH |
| *Switzerland* |  |  | 2000IU vitamin D | Knee | 137 | 70 ± 7 | 69 (50) | 0 to 4 | No |  |  |
|  |  |  | 800 IU vitamin D | Knee | 136 | 71 ± 6 | 77 (57) | 0 to 4 | No |  |  |
| Bolognesi, Belcaro (8) | INV | 54 | Standard management | Knee | 26 | 53 ± 2 | 10 (39) | No | No | Pain free walking distance, total walking distance | CRP, ERS, Fibrinogen, Plasmatic reactive oxygen metabolites, sVCAM-1 |
| *Italy* |  |  | Standard management and movardol | Knee | 28 | 52 ± 4 | 10 (36) | No | No |  |  |
| Chen, Wang (9) | OBS | 18 | Symptomatic | Knee | 18 | 54 ± 5 | 18 (100) | 2 and 3 | No | Leg extension 1RM, 30% 1RM leg extension, 70% 1RM leg extension, leg curl 1RM, 30% 1RM leg curl, 70% 1RM leg curl | IGF-1, growth hormone, blood lactate, testosterone |
| *China* |  |  |  |  |  |  |  |  |  |  |  |
| Chen, Han (10) | OBS | 5318 | OA | N/A | 698 | 49 ± 9 | 419 (60) | No | No | Appendicular Lean Mass, handgrip strength | BUN, calcium, phosphorus, triglycerides, total cholesterol, creatinine, uric acid |
| *USA* |  |  |  |  |  |  |  |  |  |  |  |
| Coppock, McNulty (11) | RCT | 59 | OA | N/A | 39 | 69 ± 6 | 30 (77) | No | No | 6-minute walk, 8ft up and go, 10 chair stands, SPPB, handgrip strength | Adiponectin, sCOMP, CRP, IL-6, TNF-α |
| *USA* |  |  |  |  |  |  |  |  |  |  |  |
| Cornish and Peeler (12) | RCT | 18 | Creatine | Knee | 9 | 58 ± 8 | 4 (44) | 2 and 3 | No | Knee extension at 0°, 45°, 90°, knee flexion at 0°, 45° and 90° | CRP, IL-1B, IL-6, s100 A8/A9, sCOMP, TNF-a |
| *Canada* |  |  | Placebo | Knee | 9 | 57 ± 7 | 4 (44) | 2 and 3 | No |  |  |
| Cuellar, Blizzard (13) | RCT | 217 | Vitamin D | knee | 104 | 64 ± 7 | 49 (47) | No | No | External oblique (contracted) thickness | 25(OH)D |
| *Australia* |  |  | Placebo | knee | 113 | 63 ± 7 | 55 (49) | No | No |  |  |
| de Almeida, Aily (14) | RCT | 61 | Circuit training | Knee | 20 | 56 ± 5 | 15 (75) | 2 (n=16)  3 (n=4) | Yes | 30 sec chair tests, 40 m walk test, knee MVIC extension, knee MVIC flexion, lean mass, stair climb | Fasting glucose, Glycemia, HDL, LDL, Total cholesterol, triglycerides |
| *Brazil* |  |  | Strength training | Knee | 21 | 55 ± 7 | 16 (76) | 2 (n=15)  3 (n=6) | Yes |  |  |
|  |  |  | Educational protocol | Knee | 20 | 54 ± 8 | 16 (80) | 2 (n=15)  3 (n=5) | Yes |  |  |
| Edd, Favre (15) | OBS | N/A | Pre-OA | Knee | 52 | 44 ± 11 | 30 (58) | No | No | Speed | TNFα |
| Australia |  |  |  |  |  |  |  |  |  |  |  |
| Erhart-Hledik, Chehab (16) | Cohort | 25 | Whole group | Knee | 25 | 59 ± 11 | 8 (32) | 1.7 + 1.1 (0-4) | No | Walking speed, knee adduction moment (first peak), knee adduction moment (impulse) | sCOMP |
| *USA* |  |  |  |  |  |  |  |  |  |  |  |
| Germanou, Chatzinikolaou (17) | OBS | 20 | Patients | Knee | 10 | 59 ± 6 | 10 (100) | >2 | Yes | Knee extensor at 90°, 120°, 150°, knee flexibility, knee flexor at 90°, 120°, 150°, knee ROM | Catalase activity, Creatine kinase activity, CRP, Exercise lactate, GPX, GSH/GSSG, IL-6, LDH, Leukocytes, LPH, Oxidized GSH, Protein carbonyls, Reduced glutathione, Resting lactate, TBARS, TAC, Uric acid |
| *Greece* |  |  |  |  |  |  |  |  |  |  |  |
| Gibson, Morrison (18) | OBS | 14 | OA | Knee | 7 | 67 ± 10 | N/A | N/A | No | Joint extension, joint flexion, quad (fatiguability 100hz 30hz,10/50hz, 20/50hz), quad force loss, quad relaxation rates (sf50, sf95), quadricep MVC, total CSA, type i diameter and total fibres, type iia diameter and total fibres, type iib diameter and total fibres | Alkali soluble muscle protein, muscle protein, synthesis; muscle protein synthesis rate, RNA, DNA, muscle protein synthesis/RNA, RNA/DNA. |
| *Scotland* |  |  | Control |  | 7 | 64 ± 10 | N/A | N/A | No |  |  |
| Gomes, Lacerda (19) | OBS | 15 | Whole group | Knee | 15 | 67 ± 4 | 15 (100) | N/A | Yes | Physical function, 6MWT, V0_2max_ | IL-6, TNF, sTNFR1, sTNFR2 |
| *Brazil* |  |  |  |  |  |  |  |  |  |  |  |
| Gudbergsen, Boesen (20) | OBS | 192 | Whole group | Knee | 192 | 63 ± 6 | 155 (81) | 3 | Yes | Quadriceps muscle strength, hamstrings muscle strength | CRP |
| *Denmark* |  |  |  |  |  |  |  |  |  |  |  |
| Hagio and Aikawa (21) | OBS | 30 | Whole Group | Hip | 30 | 66 ± 9 | 25 (83) | N/A | No | TUG, 10m walk time | Creatine Kinase, CRP, myoglobin |
| *Japan* |  |  |  |  |  |  |  |  |  |  |  |
| Han, Zeng (22) | RCT | 180 | Study | Knee | 90 | 51 ± 3 | 41 (46) | N/A | Yes | Muscle strength, ROM | IL-7, TNF-a |
| *China* |  |  | Control | Knee | 90 | 51 ± 3 | 44 (46) | N/A | Yes |  |  |
| Heidari, Javadian (23) | OBS | 67 | Whole group | Knee | 67 | 50 ± 7 | 58 (87) | 4 | Yes | Quadriceps muscle strength | 25(OH)D |
| *Iran* |  |  |  |  |  |  |  |  |  |  |  |
| Jianda, Yuxing (24) | INV | 75 | MMA with pre-emptive analgesia group | Knee | 38 | 68 ± 7 | 26 (72) | N/A | No | 2MWT, 6MWT, ROM | CRP |
| *China* |  |  | MMA without pre-emptive analgesia group | Knee | 37 | 69 ± 7 | 24 (65) | N/A | No |  |  |
| Khan and Williams (25) | RCT | 64 | Etodolac | Knee | 32 | 60 ± 8 | 19 (59) | N/A | No | Knee flexion, time to walk 15m | eosinophils, ESR, lymphocytes, Monocytes, neutrophils, platelet count, prothrombin time, red cell count, total white cell count |
| *UK* |  |  | Diclofenac | Knee | 32 | 64 ± 8 | 22 (69) | N/A | No |  |  |
| Kim, Lee (26) | RCT | 41 | Intervention | Knee | 22 | 66 ± 6 | 14 (64) | 1 to 3 | Yes | Cadence, gait cycle time, gait velocity, knee varus/valgus ROM | COMP, CRP, IGF-1, IL-1β, IL-6, IL-8, TGF-β, TNF-α, YKL-40 |
| *Republic of Korea* |  |  | Control | Knee | 19 | 65 ± 9 | 17 (90) | 1 to 3 | Yes |  |  |
| Kim, Hsu (27) | RCT | 43 | Intervention | Knee | 20 | 67 ± 6 | 10 (50) | N/A | No | SPPB balance, SPPB chair stand, SPPB gait speed | CRP, IL-6, TNFa |
| *USA* |  |  | Standard care | Knee | 23 | 67 ± 6 | 9 (39) | N/A | No |  |  |
| Kuhi, Tamm (28) | OBS | 86 | Whole group | Knee | 86 | 60 ± 5 | 43 (50) | 2 to 4 | No | 30 m fast-paced walk test, 30 s chair STS, TUG | uC2C/Creatinine |
| *Estonia* |  |  |  |  |  |  |  |  |  |  |  |
| Laslett, Quinn (29) | RCT | 133 | 4Jointz | Knee | 64 | 64 ± 10 | 35 (55) | 0 to 2 | Yes | Leg strength | IL-6, CTX-II |
| *Australia* |  |  | Placebo | Knee | 69 | 66 ± 8 | 25 (36) | 0 to 2 | Yes |  |  |
| Lee, Ro (30) | OBS | 4924 | OA | Knee | 821 | 68 ± 8 | 641 (78) | ≥2 | No | SMI, muscle mass | 25(OH)D |
| *Korea* |  |  |  |  |  |  |  |  |  |  |  |
| Levinger, Begg (31) | OBS | 24 | Whole | Knee | 24 | 69 ± 6 | 12 (50) | N/A | Yes | Isokinetic knee extension 180°, isokinetic knee extension 90°, isometric knee extension, knee absorption power, knee negative work, lower limb muscle mass, muscle quality at isokinetic 180°, muscle quality at isokinetic 90°, muscle quality at isometric° | 25(OH)D |
| *Australia* |  |  | Vitamin D insufficient | Knee | 7 | 70 ± 9 | 4 (57.1) | N/A | Yes |  |  |
|  |  |  | Vitamin D sufficient | Knee | 17 | 68 ± 5 | 8 (47) | N/A | Yes |  |  |
| Li, Wang (32) | INV | 112 | OA | Knee | 58 | 53 ± 12 | 31 (54) | 2 or 3 | No | Step length, gait speed, gait frequency | IL-1β, IL-6, IL-17, |
|  |  |  | Control | Knee | 54 | 52 ± 12 | 23 (43) | 2 or 3 | No |  |  |
| Lim, Cicuttini (33) | RCT | 304 | Atorvastatin | Knee | 151 | 56 ± 7 | 92 (61) | 0 to 2 | Yes | Quadricep muscle strength, vastus medialis CSA | Creatinine kinases, ALT, AST |
|  |  |  | Placebo | Knee | 153 | 56 ±8 | 77 (50) | 0 to 2 | Yes |  |  |
| Magrans-Courtney, Wilborn (34) | RCT | 30 | Glucosamine, chondroitin sulphate, methyl sulfonyl methane | Knee | 16 | 52 ± 10 | 16 (100) | N/A | No | Bench press 1RM, circumference, fatigue index extension - 10 reps at 180°, 15 reps at 300°, 5 reps at 60°, fatigue index flexion - 10 reps at 180°, 15 reps at 300°, 5 reps at 60°, forward lunge knee function - contact time, distance, force impulse, impact index, peak torque extension - 10 reps at 180°, 15 reps at 300°, 5 reps at 60°, peak torque flexion - 10 reps at 180°, 15 reps at 300°, 5 reps at 60°, ROM extension, ROM flexion, STS function - rising index, sway velocity, weight transfer, step up and over knee function - impact index, lift-up index, movement time, upper body endurance | ALT, AST, BUN, BUN/Creatinine Ratio, Cholesterol, creatine kinase, Cortisol, CRP, Creatinine, GGT, Glucose, Glucose/Insulin Ratio, HDL, HOMA_IR_, IL-6, Insulin, LDL, Leptin, TNF-a, Triglycerides, Uric Acid |
| *USA* |  |  | Placebo | Knee | 14 | 57 ± 7 | 14 (100) | N/A | No |  |  |
| Manoy, Yuktanandana (35) | RCT | 175 | Whole group | Knee | 175 | 65 | 158 (90) | No | Yes | Handgrip strength, knee extensor strength, timed-up and go, sit-to-stand, 6MWT | FBG, Insulin, HOMA-IR, total cholesterol, HDL, Triglycerides, LDL, 25(OH)D, calcium, phosphorus, PTH, hsCRP, IL-6, leptin, protein carbonyls |
| *Thailand* |  |  |  |  |  |  |  |  |  |  |  |
| Marriott, Chopp-Hurley (36) | RCT | 17 | Exercise | Knee | 10 | 65 ± 7 | 10 (100) | 1 to 4 | Yes | Power, strength | IL-6, CRP, TNFa |
| *Canada* |  |  | No exercise | Knee | 7 | 72 ± 11 | 7 (100) | 1 to 4 | Yes |  |  |
| McAlindon, Lavalley (37) | RCT | 146 | Vitamin D | Knee | 73 | 62 ± 8 | 49 (67) | 2 to 4 | Yes | Chair stand, 20m walk test | 25(OH)D |
| *USA* |  |  | Placebo | Knee | 73 | 63 ± 9.3 | 40 (54) | 2 to 4 | Yes |  |  |
| Messier, Loeser (38) | RCT | 24 | Exercise | Knee | 11 | 69 ± 5 | 7 (64) | N/A | No | 6MWT, ambulation frequency, ambulation intensity, ankle maximum plantarflex velocity, ankle mean velocity, ankle ROM, cadence, knee maximum extension velocity, knee mean velocity, knee ROM, loading rate-least affected knee, loading rate-most affected knee, maximum braking, maximum propulsive, mean concentric extension, mean concentric flexion, stair climb, stance time, stride length, transfer frequency, transfer intensity, vertical propulsive peak, vertical impact peak, walking velocity | IL-I β |
| *USA* |  |  | Exercise and diet | Knee | 13 | 67 ± 4 | 10 (77) | N/A | No |  |  |
| Miller, Nicklas (39) | RCT | 316 | Healthy lifestyle | Knee | 76 | 69 ± 6 | 51 (67) | N/A | No | Walking speed | Leptin |
| *USA* |  |  | Dietary weight loss | Knee | 80 | 68 ± 6 | 57 (71) | N/A | No |  |  |
|  |  |  | Exercise | Knee | 79 | 69 ± 7 | 60 (76) | N/A | No |  |  |
|  |  |  | Diet and exercise | Knee | 74 | 69 ± 7 | 55 (74) | N/A | No |  |  |
| Mundermann, King (40) | OBS | 83 | OA | Knee | 42 | 61 ± 9 | 22 (52) | 1 to 4 | Yes | 30-min walking exercise – distance, number of steps, walking speed, peak ankle dorsiflexion moment, eversion moment, peak external knee adduction moment, peak hip adduction moment, peak hip flexion moment, peak knee flexion moment, post-exercise rest - cadence, distance, number of steps | COMP |
| *USA* |  |  |  |  |  |  |  |  |  |  |  |
| Nicklas, Mychaleckyj (41) | INV | 214 | Whole | Knee | 214 | 69 ± 6 | 151 (70) | 2.23 ± 0.82 | No | Stair-climb time, walking distance | IL-6 174 G/C_CC, _CG, _GG, TNFa 238 G/A_AG, _GG, TNFa 308 G/A_AA, AG, _GG, TNFR1 +36 A/G_AA, _AG, _GG, TNFR2 +1663 A/G_AA, _AG, _GG, TNFR2 +676 T/G_GG, _GT, _TT |
| *USA* |  |  |  |  |  |  |  |  |  |  |  |
| Ngarmukos, Tanavalee (42) | OBS | 94 | 2 PRP injections | Knee | 51 | 67 | 47 (92) | 1 to 4 | Yes | Tug, 3m walking test, 5 x STS, ROM | IA-17A, Il-10, IL-13, IL-1B, IL-1RA, IL-4, IL-6, PDGF-AA, PDGF-BB, TGF-B1, TNFa, VEGF |
| *Thailand* |  |  | 4 PRP injections | Knee | 43 | 66 | 40 (93) | 1 to 4 | Yes |  |  |
| Okoro, Stewart (43) | OBS | 14 | Males | Hip | 8 | 65 ± 9 | 0 (0) | N/A | No | 30 sec STS, 6MWT, leg lean mass, MVC quadriceps, TUG | CAPN2, CTSL2, CTSL2, FOS, IL-6, LPL, PPARG, PSMA7, TNFa, |
| *UK* |  |  | Females | Hip | 6 | 61 ± 12 | 6 (100) | N/A | No |  |  |
| Ostlind, Eek (44) | RCT | 91 | Whole group | Hip or knee | 91 | 56 ± 6 | 74 (81) | N/A | No | Steps/day | CRP, ARGS, C2C, COMP |
| *Sweden* |  |  |  |  |  |  |  |  |  |  |  |
| Park, Min (45) | RCT | 81 | Control | Knee | 25 | 68 ± 4 | 25 (100) | 1 or 2 | No | Isokinetic torque 60^o^/s, skeletal muscle mass | IL-6, CRP, Resistin, TNFa |
| *South Korea* |  |  | Isometric exercise | Knee | 25 | 67 ± 5 | 25 (100) | 1 or 2 | No |  |  |
|  |  |  | Isometric exercise and electro-myo-stimulation | Knee | 25 | 66 ± 3 | 25 (100) | 1 or 2 | No |  |  |
| Peker, Peker (46) | OBS | 96 | OA | Knee | 48 | 54 ± 9 | 48 (100) | >2 | No | Leg skeletal muscle mass index, leg muscle mass index |  |
| *Turkey* |  |  | Control | Knee | 48 | 43 ± 9 | 48 (100) | >2 | No |  | Neutrophil/lymphocytes, triglycerides, LDL, HDL, triglycerides/ HDL, monocytes/  lymphocytes |
| Pérez-Piñero, Muñoz-Carrillo (47) | RCT | 55 | Experimental | Knee | 29 | 51 ± 9 | N/A | N/A | Yes | Muscle mass, TUG, knee flexor, extensor isokinetic 60^o^, 180^o^ peak torque, relative peak torque, total work, total work for 1 rep maximum, knee flexor, extensor isometric 90^o^ peak torque, relative peak torque, total work, total work for 1 rep maximum, average power | IL-1β, MMP-3, COMP |
| *Spain* |  |  | Control | Knee | 26 | 50 ± 9 | N/A | N/A | Yes |  |  |
| Perruccio, Zahid (48) | OBS | 6098 | OA | Hip, knee, hands | 3049 | 63 | 1701 (55.8) | N/A | No | TUG | CRP, HDL, triglycerides, total cholesterol, HbA1/c |
| *Canada* |  |  |  |  |  |  |  |  |  |  |  |
| Perry, Levinger (49) | OBS | 36 | OA | Knee | 19 | 70 ± 7 | 10 (53) | N/A | No | Voluntary maximal knee extensor torque - peak, voluntary maximal knee extensor torque | 3Houabain binding site content, NKA α1, NKA α2 NKA α3, NKA β1, NKA β2, NKA β3 |
| *Australia* |  |  |  |  |  |  |  |  |  |  |  |
| Petersen, Saxne (50) | RCT | 36 | Glucosamine | Knee | 12 | 62 ± 3 | 7 (58) | 2.5 ± 0.8 | Yes | Knee extension 5RM, leg press 5RM | COMP, uCTX-II |
| *Denmark* |  |  | Ibuprofen | Knee | 11 | 62 ± 5 | 7 (64) | 2.3 ± 1 | Yes |  |  |
|  |  |  | Placebo | Knee | 12 | 63 ± 5 | 7 (58) | 2.2 ± 1 | Yes |  |  |
| Pinsornsak, Kanokkangsadal (51) | RCT | 66 | Sahastara | Knee | 31 | 60 ± 7 | 28 (90) | 1 to 3 | Yes | 100m walk time test | BUN, ALP, ALT, AST, creatinine |
| *Thailand* |  |  | Diclofenac | Knee | 30 | 58 ± 8 | 27 (90) | 1 to 3 | Yes |  |  |
| Raut, Bichile (52) | RCT | 93 | Glucosamine sulphate (GS) | Knee | 26 | 59 ± 7 | 21 (60) | N/A | Yes | 50-feet walking | CTX II, ALP, Bilirubin, BUN, Creatinine, SGOT, SGPT, Haemoglobin, leucocytes count, Platelet count, TNFa-SRI, TNFa-SRII, Uric acid |
| *India* |  |  | AmrutBhallatak (ABFN02) | Knee | 33 | 58 ± 9 | 30 (77) | N/A | Yes |  |  |
|  |  |  | ABFNO2 + GS | Knee | 34 | 55 ± 8 | 27 (72) | N/A | Yes |  |  |
| Roy, de Beer (53) | RCT | 37 | Placebo | Knee | 19 | 63 ± 10 | 11 (58) | N/A | N/A | Ankle dorsiflexion strength, handgrip strength, knee extension strength, muscle fibre area, timed 30-ft walk, timed 4-step climb | Creatine, phosphocreatine, total creatine, adenosine triphosphate, creatinine |
| *Canada* |  |  | Creatine | Knee | 18 | 64 ± 10 | 9 (56) | N/A | N/A |  |  |
| Samut, Dincer (54) | RCT | 42 | Isokinetic exercise | Knee | 15 | 63 ± 8 | 14 (90) | 2 to 3 | Yes | 30-sec STS, 6MWT, peak torque isokinetic 5 maximal reciprocal concentric extension contractions for each angular velocity of 180°/s to body weight, peak torque isokinetic 5 maximal reciprocal concentric extension contractions for each angular velocity of 60°/s to body weight, peak torque isokinetic 5 maximal reciprocal concentric flexion contractions for each angular velocity of 180°/s to body weight, peak torque isokinetic 5 maximal reciprocal concentric flexion contractions for each angular velocity of 60°/s to body weight | CRP, IL-6, TNF-a |
| *Turkey* |  |  | Aerobic exercise | Knee | 14 | 58 ± 6 | 13 (90) | 2 to 3 | Yes |  |  |
|  |  |  | Control | Knee | 13 | 61 ± 9 | 12 (90) | 2 to 3 | Yes |  |  |
| Sanchez-Ramirez, van der Leeden (55) | OBS | 186 | Whole | Knee or hip | 186 | 61 ± 7 | 127 (68) | >2 | Yes | Knee muscle strength isokinetic - quadriceps - 60°, knee muscle strength isokinetic - hamstrings - 60° | CRP, ESR |
| *Amsterdam* |  |  |  |  |  |  |  |  |  |  |  |
| Sarsan, Akkaya (56) | RCT | 27 | Mud packs | Knee | 15 | 52 ± 5 | N/A | 2 to 3 | Yes | 6MWT | IGF-1, IL-6, TNFa |
| *Turkey* |  |  | Hot packs | Knee | 12 | 54 ± 8 | N/A | 2 to 3 | Yes |  |  |
| Schumacher, Pullman-Mooar (57) | RCT | 58 | Whole | Knee | 58 | 57 ± 11 | 14 (24) | 2 to 3 | Yes | Walking time | Creatinine, hsCRP, Urate |
| *USA* |  |  |  |  |  |  |  |  |  |  |  |
| Shea, Loeser (58) | Cohort | 1069 | < phylloquinone <Vitamin D | Knee | 257 | 75 ± 3 | 172 (67) | N/A | No | 400m walk, 5-STS, chair stands time, SPPB, usual 20m gait speed | Triglycerides, Vitamin D, Vitamin K |
| *USA* |  |  | < phylloquinone > Vitamin D | Knee | 459 | 75 ± 3 | 247 (54) | N/A | No |  |  |
|  |  |  | > phylloquinone < Vitamin D | Knee | 120 | 74 ± 3 | 87 (73) | N/A | No |  |  |
|  |  |  | > phylloquinone > Vitamin D | Knee | 233 | 74 ± 3 | 134 (58) | N/A | No |  |  |
|  |  |  | < Vitamin K < Vitamin D | Knee | 1351 | 61 ± 10 | 543 (40) | N/A | No |  |  |
|  |  |  | < Vitamin K > Vitamin D | Knee | 236 | 61 ± 7 | 122 (52) | N/A | No |  |  |
|  |  |  | > Vitamin K < Vitamin D | Knee | 2259 | 62 ± 9 | 1446 (64) | N/A | No |  |  |
|  |  |  | > Vitamin K > Vitamin D | Knee | 629 | 61 ± 8 | 503 (80) | N/A | No |  |  |
| Velangi, Mandalika (59) | RCT | 100 | Vitamin D3 | Knee | 25 | 30 to 65 | 0 (0) | 1 to 2 | No | 6MWT | 25(OH)D, CRP, ESR, s-COMP |
| *India* |  |  | Vitamin D3 | Knee | 25 | 30 to 65 | 25 (100) | 1 to 2 | No |  |  |
|  |  |  | Vitamin D3 and virgin coconut oil | Knee | 25 | 30 to 65 | 0 (0) | 1 to 2 | No |  |  |
|  |  |  | Vitamin D3 and virgin coconut oil | Knee | 25 | 30 to 65 | 25 (100) | 1 to 2 | No |  |  |
| Wallis, Webster (60) | RCT | 46 | Walking | Knee | 23 | 68 ± 8 | 9 (39) | 3 to 4 | No | 30 sec chair test, 40 m walk test, steps per day | Fasting glucose, HDL, LDL, total cholesterol, triglycerides |
| *Australia* |  |  | Usual care | Knee | 23 | 67 ± 7 | 11 (48) | 3 to 4 | No |  |  |
| Wang, Yang (61) | RCT | 99 | Whole body vibration exercise + quadriceps resistance exercises | Knee | 49 | 61 ± 10 | 36 (73.5) | 2 or 3 | Yes | 6-minute walk distance, active knee extension and flexion, knee extension and flexion strength, timed up and go | COMP, CTX-II, Liquesce index |
| *China* |  |  | Quadriceps resistance exercises | Knee | 50 | 62 ± 9 | 35 (70) | 2 or 3 | Yes |  |  |
| Wasser, Hendershot (62) | OBS | 38 | OA | Knee | 12 | 37 ± 7 | 0 (0) | >1 | No | Cadence, continuous relative phase (frontal/sagittal/transverse), continuous relative phase var (frontal/sagittal/transverse), gait speed, KAM impulse, KAM loading rate,), knee flexion moment, knee flexion ROM, peak ankle dorsiflexion, peak knee flexion, peak pelvic anterior tilt, peak pelvic drop (contralateral), peak pelvic drop (prosthetic), pelvic tilt ROM, pelvis ROM (frontal/sagittal/transverse), stride length, stride width, trunk ROM (frontal/sagittal/transverse), trunk-pelvis (frontal/sagittal/transverse) | C2C, COMP, CCL-11, CCL-4, CCL-5, CCL-2, CXCL-10, CTX-1, HA, IL-18, IL-1a, IL-7, INF-a, MMP-2, MMP-12, MMP-13, MMP-3, MMP-7, MMP-8, MMP-9, NTX-1, PIIANP, SDF-1, TIMP-1, TNF-a, |
| *USA* |  |  |  |  |  |  |  |  |  |  |  |
| Zertuche, Rabasa (63) | OBS | 777 | Radiographic OA | Knee | 258 | 61 ± 8 | 158 (61) | >2 | No | Mean quadriceps strength | Alkyl resorcinol - 17, - 19, - 21, - 23, - 25, - sum |
| *USA* |  |  | Symptomatic OA | Knee | 260 | 63 ± 8 | 164 (63) | >2 | No |  |  |

**1RM:** 1 repetition maximum; **S100 A8/A9:** calprotectin: **25(OH)D:** vitamin D; **25(OH)D3:** vitamin D3; **6MWT:** 6 minute walk test; **ALP:** alkaline phosphatase; **ALT:** alanine aminotransferase; **ARGS:** alanine-arginine-glycine-serine; **AST:** aspartate aminotransferase; **BUN:** blood urea nitrogen; **sC2C:** cleavage of type ii collagen by collagenases; **CAPN2:** calpain 2; **CCL-2:** chemokine (C-C Motif) ligand 2; **CCL-4:** chemokine (C-C Motif) ligand 4; **CCL-5:** chemokine (C-C Motif) ligand 5; **CCL-11:** chemokine (C-C Motif) ligand 11; s**COMP:** serum cartilage oligomeric matrix protein; **CSA:** cross-sectional area; **hs-CRP:** high-sensitivity c-reactive protein; **CRP**: c-reactive protein; **CTSL2:** cathepsin L2; **CTX-I:** c-terminal telopeptide type I collagen; **uCTX-II:** urinary c-terminal telopeptide type II collagen; **CXCL-10:** chemokine (C-X-C Motif) Ligand 10; **DNA:** deoxyribonucleic acid; **ESR:** erythrocyte sedimentation rate; **FBG:** fasting blood glucose; **FOS:** immediate early response gene; **GGT:** gamma glutamyl transferase; **GPX:** glutathione peroxidase activity; **GSH:** oxidised glutathione; **GSSG:** reduced glutathione; **GSH/GSSG:** oxidised/reduced glutathione; **HA:** hyaluronic acid; **HbA1c:** haemoglobin A1c; **HDL:** high-density lipoprotein; **HOMA_IR_:** homeostatic model assessment of insulin resistance; **IGF-1:** insulin-like growth factor-1; **IL-1α:** interleukin 1 alpha; **IL-1β:** interleukin 1 beta; **IL-1RA:** interleukin 1 receptor antagonist protein; **IL-10:** interleukin 10; **IL-12:** interleukin 12; **IL-13:** interleukin 13; **IL-17:** interleukin 17; **IL-18:** interleukin 187; **IL-2:** interleukin 2; **IL-4:** interleukin 4; **IL-5:** interleukin 5; **IL-6:** interleukin 6; **IL-7:** interleukin 7; **IL-8:** interleukin 8; **INF-α:** Interferon alpha; **INV:** intervention; **iPTH:** serum parathyroid hormone intact; **KAM:** knee adduction moment; **LDL:** low-density lipoprotein; **LDH:** lactate dehydrogenase; **LPH:** lipid hydroperoxides; **LPL:** lipoprotein lipase; **MMP-12:** serum matrix metalloproteinase 12; **MMP-13:** serum matrix metalloproteinase 13; **MMP-2:** serum matrix metalloproteinase 2; **MMP-3:** serum matrix metalloproteinase 3; **MMP-7:** serum matrix metalloproteinase 7; **MMP-8:** serum matrix metalloproteinase 8; **MMP-9:** serum matrix metalloproteinase 9; **MVIC:** maximum voluntary isometric contraction; **NKA:** sodium-potassium transporter Na-K^+^-APT pump; **NTX-1:** N-telopeptide of type I collagen; **OA:** osteoarthritis; **OBS:** observational study; **PDGF-AA:** platelet derived growth factor subunit A; **PDGF-BB:** platelet derived growth factor subunit A; **PIIANP:** N-propeptide of collagen IIA; **sPiiCP:** cartilage type ii procollagen carboxy pro-peptide; **PPARAG:** peroxisome proliferated activated receptor gamma; **PSMA7:** 20s proteasome alpha subunit 7; **PTH:** parathyroid hormone; **RCT:** randomised controlled trial; **RNA:** ribonucleic acid; **ROM:** range of motion; **SDF-1:** Stromal-cell derived factor -1; **SPPB:** short physical performance battery; **SGOT:** Glutamic Oxaloacetic Transaminase; **SGPT:** Glutamic Pyruvic Transaminase; **STS:** sit-to-stand; **TAC:** total antioxidant capacity; **TBARS:** Thio barbituric acid-reactive substances; **TGF-β:** transforming growth factor beta; **TGF-β1:** transforming growth factor beta 1; **TIMP-1:** Tissue inhibitor matrix metalloproteinase 1; **TNF-α:** tumour necrosis factor alpha; **TNF:** tumour necrosis factor; **sTNFR1:** soluble forms tumour necrosis factor alpha receptor 1; **sTNFR2:** soluble forms tumour necrosis factor alpha receptor 2; **TUG:** Timed-up and go; **sVCAM-1:** serum vascular cell adhesion molecule-1; **VEGF:** vascular endothelial growth factor; **VO_2max_:** maximum oxygen consumption; **YKL-40:** or chitinase-3-like protein.

**References**

1. Aguiar GC, Do Nascimento MR, De Miranda AS, Rocha NP, Teixeira AL, Scalzo PL. Effects of an exercise therapy protocol on inflammatory markers, perception of pain, and physical performance in individuals with knee osteoarthritis. Rheumatol Int. 2015;35(3):525-31.

2. Apparao P, S S, Ch GS, Reddy RS. Influence of Stabilization Exercises on Articular Cartilage Changes in Degenerative Tibio- Femoral Joint Disease- a Pilot Study. Asian Journal of Pharmaceutical and Clinical Research. 2017;10(4).

3. Armagan O, Serin DK, Calisir C, Dokumacioglu A, Ozgen M, Oner S, et al. Inhalation therapy of calcitonin relieves osteoarthritis of the knee. J Korean Med Sci. 2012;27(11):1405-10.

4. Arden NK, Cro S, Sheard S, Dore CJ, Bara A, Tebbs SA, et al. The effect of vitamin D supplementation on knee osteoarthritis, the VIDEO study: a randomised controlled trial. Osteoarthritis Cartilage. 2016;24(11):1858-66.

5. Azukizawa M, Ito H, Hamamoto Y, Fujii T, Morita Y, Okahata A, et al. The Effects of Well-Rounded Exercise Program on Systemic Biomarkers Related to Cartilage Metabolism. Cartilage. 2019;10(4):451-8.

6. Barker T, Henriksen VT, Rogers VE, Aguirre D, Trawick RH, Lynn Rasmussen G, et al. Vitamin D deficiency associates with gamma-tocopherol and quadriceps weakness but not inflammatory cytokines in subjects with knee osteoarthritis. Redox Biol. 2014;2:466-74.

7. Bischoff-Ferrari HA, Orav EJ, Egli A, Dawson-Hughes B, Fischer K, Staehelin HB, et al. Recovery after unilateral knee replacement due to severe osteoarthritis and progression in the contralateral knee: a randomised clinical trial comparing daily 2000 IU versus 800 IU vitamin D. RMD Open. 2018;4(2):e000678.

8. Bolognesi G, Belcaro G, Feragalli B, Cornelli U, Cotellese R, S. H, et al. Movardol(R) (N-acetylglucosamine Boswellia serrata ginger) supplementation in the management of knee osteoarthritis: preliminary results from a 6 months registry study. European Review for Medical and Pharmacological Sciences. 2016;20:5198-2504.

9. Chen Y, Wang J, Li S, Li Y. Acute effects of low load resistance training with blood flow restriction on serum growth hormone, insulin-like growth factor-1, and testosterone in patients with mild to moderate unilateral knee osteoarthritis. Heliyon. 2022;8(10):e11051.

10. Chen S, Han H, Jin J, Zhou G, Li Z. Osteoarthritis and sarcopenia-related traits: the cross-sectional study from NHANES 2011-2014 and Mendelian randomization study. J Orthop Surg Res. 2023;18(1):502.

11. Coppock JA, McNulty AL, Porter Starr KN, Holt AG, Borack MS, Kosinski AS, et al. The effects of a 6-month weight loss intervention on physical function and serum biomarkers in older adults with and without osteoarthritis. Osteoarthr Cartil Open. 2023;5(3):100376.

12. Cornish SM, Peeler JD. No effect of creatine monohydrate supplementation on inflammatory and cartilage degradation biomarkers in individuals with knee osteoarthritis. Nutr Res. 2018;51:57-66.

13. Cuellar WA, Blizzard L, Hides JA, Callisaya ML, Jones G, Cicuttini F, et al. Vitamin D supplements for trunk muscle morphology in older adults: secondary analysis of a randomized controlled trial. J Cachexia Sarcopenia Muscle. 2019;10(1):177-87.

14. de Almeida AC, Aily JB, Pedroso MG, Goncalves GH, Pastre CM, Mattiello SM. Reductions of cardiovascular and metabolic risk factors after a 14-week periodized training model in patients with knee osteoarthritis: a randomized controlled trial. Clin Rheumatol. 2021;40(1):303-14.

15. Edd SN, Favre J, Blazek K, Omoumi P, Asay JL, Andriacchi TP. Altered gait mechanics and elevated serum pro-inflammatory cytokines in asymptomatic patients with MRI evidence of knee cartilage loss. Osteoarthritis Cartilage. 2017;25(6):899-906.

16. Erhart-Hledik JC, Chehab EF, Asay JL, Favre J, Chu CR, Andriacchi TP. Longitudinal changes in tibial and femoral cartilage thickness are associated with baseline ambulatory kinetics and cartilage oligomeric matrix protein (COMP) measures in an asymptomatic aging population. Osteoarthritis Cartilage. 2021;29(5):687-96.

17. Germanou EI, Chatzinikolaou A, Malliou P, Beneka A, Jamurtas AZ, Bikos C, et al. Oxidative stress and inflammatory responses following an acute bout of isokinetic exercise in obese women with knee osteoarthritis. Knee. 2013;20(6):581-90.

18. Gibson JN, Morrison WL, Scrimgeour CM, Smith K, Stoward PJ, Rennie MJ. Effects of therapeutic percutaneous electrical stimulation of atrophic human quadriceps on muscle composition, protein synthesis and contractile properties. Eur J Clin Invest. 1989;19(2):206-12.

19. Gomes WF, Lacerda AC, Mendonca VA, Arrieiro AN, Fonseca SF, Amorim MR, et al. Effect of aerobic training on plasma cytokines and soluble receptors in elderly women with knee osteoarthritis, in response to acute exercise. Clin Rheumatol. 2012;31(5):759-66.

20. Gudbergsen H, Boesen M, Lohmander LS, Christensen R, Henriksen M, Bartels EM, et al. Weight loss is effective for symptomatic relief in obese subjects with knee osteoarthritis independently of joint damage severity assessed by high-field MRI and radiography. Osteoarthritis Cartilage. 2012;20(6):495-502.

21. Hagio K, Aikawa K. Minimally invasive surgery supercapsular percutaneously-assisted total hip (SuperPath) arthroplasty. Acta Biomed. 2023;94(3):e2023069.

22. Han J, Zeng Z, Pei F, Zheng T. An implementatrion study of pariarticular knee osteotomy in the treatment of knee osteoarthritis. Am J Transl Res. 2021;13(5):4771-9.

23. Heidari B, Javadian Y, Babaei M, Ghahari BY. Restorative effect of vitamin d dificiency on knee pain and quadriceps muscle strength in knee osteoarthritis. Acta Med Iran. 2015;53(8):466-70.

24. Jianda X, Yuxing Q, Yi G, Hong Z, Libo P, Jianning Z. Impact of Preemptive Analgesia on inflammatory responses and Rehabilitation after Primary Total Knee Arthroplasty: A Controlled Clinical Study. Sci Rep. 2016;6:30354.

25. Khan FM, Williams PI. Double-blind comparison of etodolac SR and diclofenac SR in the treatment of patients with degenerative joint disease of the knee. Curr Med Res Opin. 1992;13(1):1-12.

26. Kim M, Lee KH, Han SH, Lee SJ, Kim CG, Choi JH, et al. Effect of Peat Intervention on Pain and Gait in Patients with Knee Osteoarthritis: A Prospective, Double-Blind, Randomized, Controlled Study. Evid Based Complement Alternat Med. 2020;2020:8093526.

27. Kim S, Hsu FC, Groban L, Williamson J, Messier S. A pilot study of aquatic prehabilitation in adults with knee osteoarthritis undergoing total knee arthroplasty - short term outcome. BMC Musculoskelet Disord. 2021;22(1):388.

28. Kuhi L, Tamm AE, Kumm J, Järv K, Märtson A, Tamm AO, et al. Associations of Urinary Collagen II Neoepitope C2C with Total Knee Replacement Outcomes: Is OA a Systemic Disease in Rapidly Progressive Cases? Applied Sciences. 2021;12(1).

29. Laslett LL, Quinn SJ, Darian-Smith E, Kwok M, Fedorova T, Korner H, et al. Treatment with 4Jointz reduces knee pain over 12 weeks of treatment in patients with clinical knee osteoarthritis: a randomised controlled trial. Osteoarthritis Cartilage. 2012;20(11):1209-16.

30. Lee SY, Ro HJ, Chung SG, Kang SH, Seo KM, Kim DK. Low Skeletal Muscle Mass in the Lower Limbs Is Independently Associated to Knee Osteoarthritis. PLoS One. 2016;11(11):e0166385.

31. Levinger P, Begg R, Sanders KM, Nagano H, Downie C, Petersen A, et al. The effect of vitamin D status on pain, lower limb strength and knee function during balance recovery in people with knee osteoarthritis: an exploratory study. Arch Osteoporos. 2017;12(1):83.

32. Li Q, Wang H, Wang D. Effects of high tibial osteotomy combined with arthroscopy on local inflammation degree and gait activity index in patients with medial knee osteoarthritis. Pak J Med Sci. 2023;39(1):161-5.

33. Lim YZ, Cicuttini FM, Wluka AE, Jones G, Hill CL, Forbes AB, et al. Effect of atorvastatin on skeletal muscles of patients with knee osteoarthritis: Post-hoc analysis of a randomised controlled trial. Front Med (Lausanne). 2022;9:939800.

34. Magrans-Courtney T, Wilborn C, Rasmussen C, Ferreira M, Greenwood L, Campbell B, et al. Effects of diet type and supplementation of glucosamine, chondroitin, and MSM on body composition, functional status, and markers of health in women with knee osteoarthritis initiating a resistance-based exercise and weight loss program. J Int Soc Sports Nutr. 2011;8(1):8.

35. Manoy P, Yuktanandana P, Tanavalee A, Anomasiri W, Ngarmukos S, Tanpowpong T, et al. Vitamin D Supplementation Improves Quality of Life and Physical Performance in Osteoarthritis Patients. Nutrients. 2017;9(8).

36. Marriott K, Chopp-Hurley J, Loukov D, Karampatos S, Kuntz AB, Wiebenga EG, et al. Muscle strength gains after strengthening exercise explained by reductions in serum inflammation in women with knee osteoarthritis. Clin Biomech (Bristol, Avon). 2021;86:105381.

37. McAlindon TE, Lavalley M, Schneider E, Nuite M, Lee JY, Price LL, et al. Effects of vitamine d supplementation on progression of knee pain and cartilage volume loss in patients with symptomatic osteoarthritis. JAMA. 2013;309(2):155-62.

38. Messier SP, Loeser RF, Mitchell MN, Valle G, Morgan TP, Rejeski WJ, et al. Exercise and weight loss in obese older adults with knee osteoarthritis: a preliminary study. J Am Geriatr Soc. 2000;48(9):1062-72.

39. Miller GD, Nicklas BJ, Davis CC, Ambrosius WT, Loeser RF, Messier SP. Is serum leptin related to physical function and is it modifiable through weight loss and exercise in older adults with knee osteoarthritis? Int J Obes Relat Metab Disord. 2004;28(11):1383-90.

40. Mundermann A, King KB, Smith RL, Andriacchi TP. Change in serum COMP concentration due to ambulatory load is not related to knee OA status. J Orthop Res. 2009;27(11):1408-13.

41. Nicklas BJ, Mychaleckyj J, Kritchevsky S, Palla S, Lange LA, Lange EM, et al. Physical function and its response to exercise: Associations with cytokine gene variation in older adults with knee osteoarthritis. J Gerontol a-Biol. 2005;60(10):1292-8.

42. Ngarmukos S, Tanavalee C, Amarase C, Phakham S, Mingsiritham W, Reantragoon R, et al. Two or four injections of platelet-rich plasma for osteoarthritic knee did not change synovial biomarkers but similarly improved clinical outcomes. Sci Rep. 2021;11(1):23603.

43. Okoro T, Stewart C, Al-Shanti N, Lemmey A, Maddison P, Andrew JG. Objective Function, Lean Mass, and Associated Genetic Adaptations of the Operated Leg Following Total Hip Arthroplasty. Journal of Musculoskeletal Research. 2015;18(02).

44. Ostlind E, Eek F, Stigmar K, Sant'Anna A, Ekvall Hansson E, Struglics A. Associations Between Physical Activity, Self-reported Joint Function, and Molecular Biomarkers in Working Age Individuals With Hip and/or Knee Osteoarthritis. Clin Med Insights Arthritis Musculoskelet Disord. 2022;15:11795441221081063.

45. Park S, Min S, Park SH, Yoo J, Jee YS. Influence of Isometric Exercise Combined With Electromyostimulation on Inflammatory Cytokine Levels, Muscle Strength, and Knee Joint Function in Elderly Women With Early Knee Osteoarthritis. Front Physiol. 2021;12:688260.

46. Peker G, Peker DM, Köseoğlu R, Gül O. Do body composition, hemogram and lipids differ between obese women with and without gonarthrosis? Annals of Clinical and Analytical Medicine. 2023;14(05):399-403.

47. Pérez-Piñero S, Muñoz-Carrillo JC, Victoria-Montesinos D, García-Muñoz AM, Ávila-Gandía V, López-Román FJ. Effectiveness of a Cucumber Extract Supplement on Articular Pain in Patients with Knee Osteoarthritis: A Randomized Double-Blind Controlled Clinical Trial. Applied Sciences. 2022;13(1).

48. Perruccio AV, Zahid S, Yip C, Power JD, Canizares M, Heckman GA, et al. Cardiovascular Risk Profile and Osteoarthritis-Considering Sex and Multisite Joint Involvement: A Canadian Longitudinal Study on Aging. Arthritis Care Res (Hoboken). 2023;75(4):893-901.

49. Perry BD, Levinger P, Serpiello FR, Caldow MK, Cameron-Smith D, Bartlett JR, et al. The effects of osteoarthritis and age on skeletal muscle strength, Na+-K+-ATPase content, gene and isoform expression. J Appl Physiol (1985). 2013;115(10):1443-9.

50. Petersen SG, Saxne T, Heinegard D, Hansen M, Holm L, Koskinen S, et al. Glucosamine but not ibuprofen alters cartilage turnover in osteoarthritis patients in response to physical training. Osteoarthritis Cartilage. 2010;18(1):34-40.

51. Pinsornsak P, Kanokkangsadal P, Itharat A. The Clinical Efficacy and Safety of the Sahastara Remedy versus Diclofenac in the Treatment of Osteoarthritis of the Knee: A Double-Blind, Randomized, and Controlled Trial. Evid Based Complement Alternat Med. 2015;2015:103046.

52. Raut A, Bichile L, Chopra A, Patwardhan B, Vaidya A. Comparative study of amrutbhallataka and glucosamine sulphate in osteoarthritis: Six months open label randomized controlled clinical trial. J Ayurveda Integr Med. 2013;4(4):229-36.

53. Roy BD, de Beer J, Harvey D, Tarnopolsky MA. Creatine monohydrate supplementation does not improve functional recovery after total knee arthroplasty. Arch Phys Med Rehabil. 2005;86(7):1293-8.

54. Samut G, Dincer F, Ozdemir O. The effect of isokinetic and aerobic exercises on serum interleukin-6 and tumor necrosis factor alpha levels, pain, and functional activity in patients with knee osteoarthritis. Mod Rheumatol. 2015;25(6):919-24.

55. Sanchez-Ramirez DC, van der Leeden M, van der Esch M, Roorda LD, Verschueren S, van Dieen JH, et al. Elevated C-reactive protein is associated with lower increase in knee muscle strength in patients with knee osteoarthritis: a 2-year follow-up study in the Amsterdam Osteoarthrititis (AMS-OA) cohort. Arthritis Res Ther. 2014;16(3):R123.

56. Sarsan A, Akkaya N, Ozgen M, Yildiz N, Atalay NS, Ardic F. Comparing the efficacy of mature mud pack and hot pack treatments for knee osteoarthritis. J Back Musculoskelet Rehabil. 2012;25(3):193-9.

57. Schumacher HR, Pullman-Mooar S, Gupta SR, Dinnella JE, Kim R, McHugh MP. Randomized double-blind crossover study of the efficacy of a tart cherry juice blend in treatment of osteoarthritis (OA) of the knee. Osteoarthritis Cartilage. 2013;21(8):1035-41.

58. Shea MK, Loeser RF, McAlindon TE, Houston DK, Kritchevsky SB, Booth SL. Association of Vitamin K Status Combined With Vitamin D Status and Lower-Extremity Function: A Prospective Analysis of Two Knee Osteoarthritis Cohorts. Arthritis Care Res (Hoboken). 2018;70(8):1150-9.

59. Velangi M, Mandalika S, Shukla S, Pradhan V. Effect of Vitamin D3 and Virgin Coconut Oil on Cartilage Degeneration, Inflammation and Functional Abilities in Early Knee Osteoarthritis. Functional Foods in Health and Disease. 2019;9(10).

60. Wallis JA, Webster KE, Levinger P, Singh PJ, Fong C, Taylor NF. A walking program for people with severe knee osteoarthritis did not reduce pain but may have benefits for cardiovascular health: a phase II randomised controlled trial. Osteoarthritis Cartilage. 2017;25(12):1969-79.

61. Wang P, Yang L, Liu C, Wei X, Yang X, Zhou Y, et al. Effects of Whole Body Vibration Exercise associated with Quadriceps Resistance Exercise on functioning and quality of life in patients with knee osteoarthritis: a randomized controlled trial. Clin Rehabil. 2016;30(11):1074-87.

62. Wasser JG, Hendershot BD, Acasio JC, Krupenevich RL, Pruziner AL, Miller RH, et al. A Comprehensive, Multidisciplinary Assessment for Knee Osteoarthritis Following Traumatic Unilateral Lower Limb Loss in Service Members. Mil Med. 2022.

63. Zertuche JP, Rabasa G, Lichtenstein AH, Matthan NR, Nevitt M, Torner J, et al. Alkylresorcinol, a biomarker for whole grain intake, and its association with osteoarthritis: the MOST study. Osteoarthritis Cartilage. 2022;30(10):1337-43.

| **Section and Topic** | **Item #** | **Checklist item** | **Location where item is reported** |
| --- | --- | --- | --- |
| **TITLE** | | |  |
| Title | 1 | Identify the report as a systematic review. | Pg 1 |
| **ABSTRACT** | | |  |
| Abstract | 2 | See the PRISMA 2020 for Abstracts checklist. | Pg 2 |
| **INTRODUCTION** | | |  |
| Rationale | 3 | Describe the rationale for the review in the context of existing knowledge. | Pg 3 |
| Objectives | 4 | Provide an explicit statement of the objective(s) or question(s) the review addresses. | Pg 3 |
| **METHODS** | | |  |
| Eligibility criteria | 5 | Specify the inclusion and exclusion criteria for the review and how studies were grouped for the syntheses. | Pg 4 |
| Information sources | 6 | Specify all databases, registers, websites, organisations, reference lists and other sources searched or consulted to identify studies. Specify the date when each source was last searched or consulted. | Pg 4 |
| Search strategy | 7 | Present the full search strategies for all databases, registers and websites, including any filters and limits used. | Supp Material |
| Selection process | 8 | Specify the methods used to decide whether a study met the inclusion criteria of the review, including how many reviewers screened each record and each report retrieved, whether they worked independently, and if applicable, details of automation tools used in the process. | Pg 4 |
| Data collection process | 9 | Specify the methods used to collect data from reports, including how many reviewers collected data from each report, whether they worked independently, any processes for obtaining or confirming data from study investigators, and if applicable, details of automation tools used in the process. | Pg 4 |
| Data items | 10a | List and define all outcomes for which data were sought. Specify whether all results that were compatible with each outcome domain in each study were sought (e.g. for all measures, time points, analyses), and if not, the methods used to decide which results to collect. | Pg 5 |
|  | 10b | List and define all other variables for which data were sought (e.g. participant and intervention characteristics, funding sources). Describe any assumptions made about any missing or unclear information. | Pg 5 |
| Study risk of bias assessment | 11 | Specify the methods used to assess risk of bias in the included studies, including details of the tool(s) used, how many reviewers assessed each study and whether they worked independently, and if applicable, details of automation tools used in the process. | Pg 4 |
| Effect measures | 12 | Specify for each outcome the effect measure(s) (e.g. risk ratio, mean difference) used in the synthesis or presentation of results. | Pg 5 |
| Synthesis methods | 13a | Describe the processes used to decide which studies were eligible for each synthesis (e.g. tabulating the study intervention characteristics and comparing against the planned groups for each synthesis (item #5)). | Pg 5 |
|  | 13b | Describe any methods required to prepare the data for presentation or synthesis, such as handling of missing summary statistics, or data conversions. | Pg 5 |
|  | 13c | Describe any methods used to tabulate or visually display results of individual studies and syntheses. | Pg 5 |
|  | 13d | Describe any methods used to synthesize results and provide a rationale for the choice(s). If meta-analysis was performed, describe the model(s), method(s) to identify the presence and extent of statistical heterogeneity, and software package(s) used. | Pg 5 |
|  | 13e | Describe any methods used to explore possible causes of heterogeneity among study results (e.g. subgroup analysis, meta-regression). | Pg 5 |
|  | 13f | Describe any sensitivity analyses conducted to assess robustness of the synthesized results. | Pg 5 |
| Reporting bias assessment | 14 | Describe any methods used to assess risk of bias due to missing results in a synthesis (arising from reporting biases). | Pg 5 |
| Certainty assessment | 15 | Describe any methods used to assess certainty (or confidence) in the body of evidence for an outcome. | N/A |
| **RESULTS** | | |  |
| Study selection | 16a | Describe the results of the search and selection process, from the number of records identified in the search to the number of studies included in the review, ideally using a flow diagram. | Fig 1 / Pg 6 |
|  | 16b | Cite studies that might appear to meet the inclusion criteria, but which were excluded, and explain why they were excluded. | Fig 1 / Pg 6 |
| Study characteristics | 17 | Cite each included study and present its characteristics. | Table 3 |
| Risk of bias in studies | 18 | Present assessments of risk of bias for each included study. | Table 2 |
| Results of individual studies | 19 | For all outcomes, present, for each study: (a) summary statistics for each group (where appropriate) and (b) an effect estimate and its precision (e.g. confidence/credible interval), ideally using structured tables or plots. | Fig 2 /  Table 4 |
| Results of syntheses | 20a | For each synthesis, briefly summarise the characteristics and risk of bias among contributing studies. | Fig 2 / Pg 6 |
|  | 20b | Present results of all statistical syntheses conducted. If meta-analysis was done, present for each the summary estimate and its precision (e.g. confidence/credible interval) and measures of statistical heterogeneity. If comparing groups, describe the direction of the effect. | Fig 2 / Pg 6/7 |
|  | 20c | Present results of all investigations of possible causes of heterogeneity among study results. | N/A |
|  | 20d | Present results of all sensitivity analyses conducted to assess the robustness of the synthesized results. | N/A |
| Reporting biases | 21 | Present assessments of risk of bias due to missing results (arising from reporting biases) for each synthesis assessed. | N/A |
| Certainty of evidence | 22 | Present assessments of certainty (or confidence) in the body of evidence for each outcome assessed. | N/A |
| **DISCUSSION** | | |  |
| Discussion | 23a | Provide a general interpretation of the results in the context of other evidence. | Pg 8-11 |
|  | 23b | Discuss any limitations of the evidence included in the review. | Pg 8-11 |
|  | 23c | Discuss any limitations of the review processes used. | Pg 8-11 |
|  | 23d | Discuss implications of the results for practice, policy, and future research. | Pg 8-11 |
| **OTHER INFORMATION** | | |  |
| Registration and protocol | 24a | Provide registration information for the review, including register name and registration number, or state that the review was not registered. | Pg 4 |
|  | 24b | Indicate where the review protocol can be accessed, or state that a protocol was not prepared. | Pg 4 |
|  | 24c | Describe and explain any amendments to information provided at registration or in the protocol. | N/A |
| Support | 25 | Describe sources of financial or non-financial support for the review, and the role of the funders or sponsors in the review. | Pg 11 |
| Competing interests | 26 | Declare any competing interests of review authors. | Pg 11 |
| Availability of data, code and other materials | 27 | Report which of the following are publicly available and where they can be found: template data collection forms; data extracted from included studies; data used for all analyses; analytic code; any other materials used in the review. | Pg 11 |

*From:*  Page MJ, McKenzie JE, Bossuyt PM, Boutron I, Hoffmann TC, Mulrow CD, et al. The PRISMA 2020 statement: an updated guideline for reporting systematic reviews. BMJ 2021;372:n71. doi: 10.1136/bmj.n71

For more information, visit: <http://www.prisma-statement.org/>
